# Eco-biochemical responses, phytoremediation potential and molecular genetic analysis of *Alhagi maurorum* grown in metal-contaminated soils

**DOI:** 10.1186/s12870-022-03768-6

**Published:** 2022-08-01

**Authors:** Yasmin M. Heikal, Mohamed A. El-Esawi, Ravi Naidu, Maha M. Elshamy

**Affiliations:** 1 Botany Department, Faculty of Science, Mansoura University, Mansoura, 35516 Egypt; 2 Botany Department, Faculty of Science, Tanta University, Tanta, 31527 Egypt; 3 Global Centre for Environmental Remediation (GCER), College of Engineering, Science and Environment, The University of Newcastle, Callaghan, NSW 2308 Australia

**Keywords:** *Alhagi maurorum*, Sequence-related amplified polymorphism, Inter simple sequence repeats, Heavy metals, Genetic variation, Antioxidants, gene expression, Eco-biochemical traits

## Abstract

**Background:**

*Alhagi maurorum* Medik. (camelthorn) is a dominant desert plant indigenous in various habitats, including the Western Desert of Egypt. The plant is especially prevalent in and around economic iron ore deposits. Nutrient and heavy metal levels in *A. maurorum* tissues and soil samples were assessed to identify associations between heavy metal levels in plants and soil. The objective was to evaluate this species as an indicator of heavy metal pollution. Photosynthetic pigments, protein, proline, alkaloids, flavonoids, 2,2-diphenyl-1-picrylhydrazylscavenging, reduced glutathione, malondialdehyde, antioxidant enzymes, and stress-related gene expression were assessed to determine their functional roles in metal stress adaptation in ultra- and molecular structure. Additionally, the molecular genetic variation in *A. maurorum* samples was assessed using co-dominant sequence-related amplified polymorphism (SRAP) and inter simple sequence repeats (ISSR).

**Results:**

A substantial difference in enzymatic and non-enzymatic antioxidants of *A. maurorum* was observed in samples collected from three sites. *A. maurorum* is suited to the climate in mineralized regions. Morphologically, the stem shows spines, narrow leaves, and a reduced shoot system. Anatomically, modifications included a cuticle coating on leaves and stems, sunken stomata, a compact epidermis, and a thick cortex. Significant anatomical-physiological differences were observed with varying heavy metal soil content, antioxidative enzyme activities increased as a tolerance strategy, and glutathione levels decreased in response to heavy metal toxicity. Heavy metal accumulation also affected the expression of stress-related genes. The highest levels of expression of *GST*, *G6PDH*, *6PGD*, *nitrate reductase 1*, and *sulfate transporter* genes were found in plants collected from site A1. However, *auxin-induced protein* exhibited its highest expression in plants collected from A2. Six SRAP combinations yielded 25 scoreable markers with a polymorphism rate of 64%, and 5 ISSR markers produced 11 bands with a polymorphism rate of 36.36% for three *A. maurorum* genotypes. The ME1xEM7 primer combinations provided the most polymorphic information content and resolving power, making it the most useful primer for differentiating *A. maurorum* genotypes. SRAP markers exhibited a higher diversity index (0.24) than ISSR markers (0.16).

**Conclusions:**

*A. maurorum* displayed adaptive characteristics for heavy metal sequestration from mining site soils and is proposed as a strong candidate for phytoremediation.

## Background

Heavy metals (HMs) are released by mining and metallurgical operations and thus, cause a threat to the environment. Fauna and flora sequester large concentrations of HMs in these environments [1]. Egypt has a long history of mining, which dates to predynastic times. The country has significant mineral wealth, with iron, phosphates, gold, and salts being the most important in terms of production volume. Mining of economic iron ore deposits at El-Wahat El-Bahariya extracts ores with an average iron content of 47.6% [2]. Groundwater at the Bahariya Oasis is used to help meet water demand for local agriculture, yet this resource shows iron levels as high as 40–60 mg/L on average. Such levels are substantially higher than the permissible limit for agricultural purposes (5 mg/L) [3].

Plants can be used for biological control of soil, air, and water contamination [4, 5]. Phytoremediation encompasses all plant-based bioremediation technologies [6]. Phytoremediation of soil polluted with HMs is recognized as a cost effective and environmentally sustainable clean-up technology. This approach employs plants to minimize, eliminate, immobilize environmental pollutants to restore sites for alternative private or public use [7]. *Alhagi maurorum* Medik., Fabaceae (commonly referred to as camelthorn, camelthorn-bush, Caspian manna, or Persian mannaplant) is an indigenous plant in the deserts of Persia, Egypt, Syria, Pakistan, and India but has been introduced to other parts of the world [8]. The plant notably grows well in iron mining areas and tolerates salty, sandy, rocky, and dry soils. *A. maurorum* is purgative, diaphoretic, and expectorant used in folk medicine to treat piles, migraines, warts, and rheumatism [9].

HMs accumulate in various plant parts depending on species, metals, and soil conditions [10, 11]. The availability of HMs for plant uptake is affected by soil parameters, such as organic matter, pH, and cation exchange capacity [12]. Some metals, such as copper (Cu), cobalt (Co), manganese (Mn), iron (Fe), zinc (Zn), and molybdenum (Mo) are essential for plant metabolism and growth but may be poisonous at supra-optimal levels [13]. HM phytotoxicity can be caused by a variety of cellular and molecular mechanisms, such as blocking functional groups of metabolic molecules, inactivating enzymes, displacing or substituting for essential components, and disrupting membrane integrity [14]. Hyperaccumulating plants exhibit metallic or metalloid elements in aerial tissues to levels that exceed usual physiological requirements for most plant species [15]. HM concentrations in tissues of such plants are commonly used to monitor environmental contamination resulting from iron mining [16].

Abiotic stress causes plants to produce reactive oxygen species (ROS), which can disrupt cell biomolecules [17]. Peroxidation of membrane lipids is a primary mechanism for ROS-induced toxicity and may be assessed by measuring malondialdehyde (MDA) levels. Tolerant plants develop antioxidant mechanisms involving antioxidant enzymes, such as catalase (CAT), superoxide dismutase (SOD), ascorbate peroxidase (APX), metabolites (phenolics and carotenoids), and expression of stress-tolerance genes to counteract the negative impacts of ROS [18]. Forest trees grown in lead-contaminated soil with different levels of water stress exhibited significantly increased peroxidase (POD) and SOD levels [19]. Secondary metabolites have a key role in plant growth and development under normal conditions and are also defense and tolerance mechanisms in response to environmental stress [20]. Further, glutathione (GSH) has a role in cellular protection against xenobiotics, oxyradicals, and metal cations [21]. GSH mitigates metal toxicity in cells by chelating metal ions and protects macromolecules by trapping free radicals [22]. ROS production in chloroplasts increases in response to environmental stress, and GSH involvement in antioxidative defenses justifies its use as a stress indicator [23].

HMs are often translocated and deposited in the root cell walls [24]. Exodermis and endodermis are important barriers to the absorption of metal ions [25]. Further, metal absorption and tolerance are affected by changes in leaf tissues and studying such changes may produce a more complete understanding of associated processes [26]. Awmack and Lock [27] found that the xylem of *A. maurorum* is well-formed, supporting the conduction of significant amount of water from root to stem. Also, the parenchymatous pith of roots retains moisture as a xerophytic adaptation.

Various molecular markers are applied alone or in combination to evaluate genetic diversity and phylogenetics in plant species [28, 29]. The sequence-related amplified polymorphism (SRAP) approach is a highly repeatable DNA sequencing method [30], used in various applications, including genetic diversity assessment [31]. SRAP markers are selected over other molecular markers to take advantage of their simplicity, access to several co-dominant markers, and targeting of open reading frames [31, 32]. SRAP markers were successfully utilized to analyze genetic diversity and population structures of numerous species, including *Brassica juncea* (brown mustard) [33], *Melia* species [34], and *Carthamus tinctorius* L (Safflower) [35].

Dominant inter simple sequence repeat (ISSR) markers have several advantages, including the ability to detect high levels of polymorphism at a low cost and simple method with an outstanding stability and repeatability [36–38]. ISSR marker technology is successfully used in genetic diversity studies, DNA fingerprinting, and germplasm assessment in plants, including *Tuberaria major* [39], *A. maurorum* [40], and *Alhagi* sp. [38].

The present study evaluated molecular genetic variation and eco-biochemical and anatomical attributes of *A. maurorum* growing in iron mining areas at El-Gedida in Egypt. The assessment also focused on the possible utility of this species for phytoremediation. Findings provide a deeper understanding of HM accumulation in various tissues of *A. maurorum*.

## Results

### Analysis of soil and heavy metal availability

Table 1 indicates the mean values of the characteristics of topsoil samples from iron mining studied sites (A1, A2 and A3). pH of soils tended toward alkaline (7.73–8.98). Organic matter values ranged between 3.0 and 3.5%, indicating relatively poor content. EC ranged from 0.40 (A1) to 7.88 (A3) mmho/cm. The latter soil is from Harrah Oasis, where high salinity is observed. The highest value for K (1611.0 ppm) was recorded at A1, and the highest concentrations for total N (839.0 ppm) and total P (163.0 ppm) were recorded at A2. EC and pH are the most important factors since HMs are less available under alkaline conditions.

**Table 1:** Geochemical characteristics of soil collected from the studied area

Parameters	A1	A2	A3	*F*-value
**pH**	**7.73 ± 0.223**^**a**^	**7.79 ± 0.270**^**a**^	**8.98 ± 0.207**^**b**^	**9.003**
**EC (mmho/cm)**	**0.40 ± 0.006**^**a**^	**1.22 ± 0.027**^**b**^	**7.88 ± 0.132**^**c**^	**2771.371**
**OC%**	**3.00 ± 0.069**^**a**^	**3.50 ± 0.121**^**a**^	**3.20 ± 0.185**^**a**^	**3.543**
**N (ppm)**	**816.0 ± 28.267**^**b**^	**839.0 ± 24.220**^**b**^	**224.0 ± 9.053**^**a**^	**248.442**
**P**	**87.00 ± 3.014**^**b**^	**163.00 ± 4.705**^**c**^	**29.00 ± 0.837**^**a**^	**424.377**
**K**	**1611.0 ± 46.506**^**c**^	**794.0 ± 18.337**^**b**^	**346.0 ± 9.988**^**a**^	**474.922**
**Fe (mg/kg)**	**24.50 ± 0.566**^**c**^	**10.50 ± 0.303**^**b**^	**6.34 ± 0.220**^**a**^	**590.005**
**Zn**	**3.06 ± 0.088**^**c**^	**2.30 ± 0.066**^**b**^	**1.26 ± 0.036**^**a**^	**180.992**
**Mn**	**8.02 ± 0.185**^**b**^	**17.40 ± 0.603**^**c**^	**5.38 ± 0.124**^**a**^	**289.835**
**Cu**	**17.09 ± 0.493**^**c**^	**6.40 ± 0.222**^**b**^	**3.84 ± 0.133**^**a**^	**477.687**
**Co**	**8.60 ± 0.248**^**b**^	**18.30 ± 0.423**^**c**^	**1.80 ± 0.0520**^**a**^	**849.135**
**Ni**	**32.10 ± 0.927**^**c**^	**28.50 ± 0.987**^**b**^	**6.90 ± 0.199**^**a**^	**297.525**
**Cd**	**9.22 ± 0.213**^**b**^	**10.89 ± 0.314**^**c**^	**1.12 ± 0.019**^**a**^	**566.801**
**Cr**	**6.90 ± 0.199**^**a**^	**7.91 ± 0.228**^**a**^	**7.82 ± 0.316**^**a**^	**4.892**
**Pb**	**33.00 ± 0.762**^**b**^	**45.00 ± 1.299**^**c**^	**2.00 ± 0.058**^**a**^	**650.193**
**Mo**	**29.90 ± 0.691**^**b**^	**34.50 ± 0.797**^**c**^	**7.00 ± 0.162**^**a**^	**572.109**
**B**	**2.50 ± 0.087**^**b**^	**3.60 ± 0.083**^**c**^	**1.40 ± 0.032**^**a**^	**234.840**

The results are recorded as Mean of triplicates ± Standard Error (SE). Different superscript letters refer to significant differences at (*P* < 0.05) level

HM levels in the soil showed significantly higher levels of Zn, Ni, Cu, and Fe in soil from the iron ore site (A1). Bioavailability of HMs for composite soil samples from the iron mining site (A1) were, in descending order, Pb > Ni > Mo > Fe > Cu > Cd > Co > Mn > Cr > Zn > B. At A2, the order was Pb > Mo > Ni > Co > Mn > Cd > Fe > Cr > Cu > Zn, and for sandy soil at A3, which exhibited the lowest levels of HMs, Cr > Mo > Ni > Fe > Mn > Cu > Pb > Co > B > Zn > Cd (Table 1).

Pearson’s correlation coefficients were strongly positive among metals associated with iron ore, regardless of concentration: 0.99 for K-Fe, 0.91 for Fe-Zn and 0.99 for Fe-Cu. Correlation coefficients among particular metals, EC, and pH were strongly negative: − 0.99 for pH-total nitrogen (N) and pH-Ni, − 0.96 for EC-Cd and EC-Mo, − 0.94 for EC-Zn, and − 0.93 for EC-Pb. Total nitrogen (N) was strongly and positively correlated with several HMs: 0.98 for N-Ni, N-Cd and N-Mo; 0.96 for N-Pb; 0.88 for N-Zn; and 0.86 for N-B. All samples were poor in organic matter, and a weak correlation between organic matter and HM content might exist (Table 2).

**Table 2:**
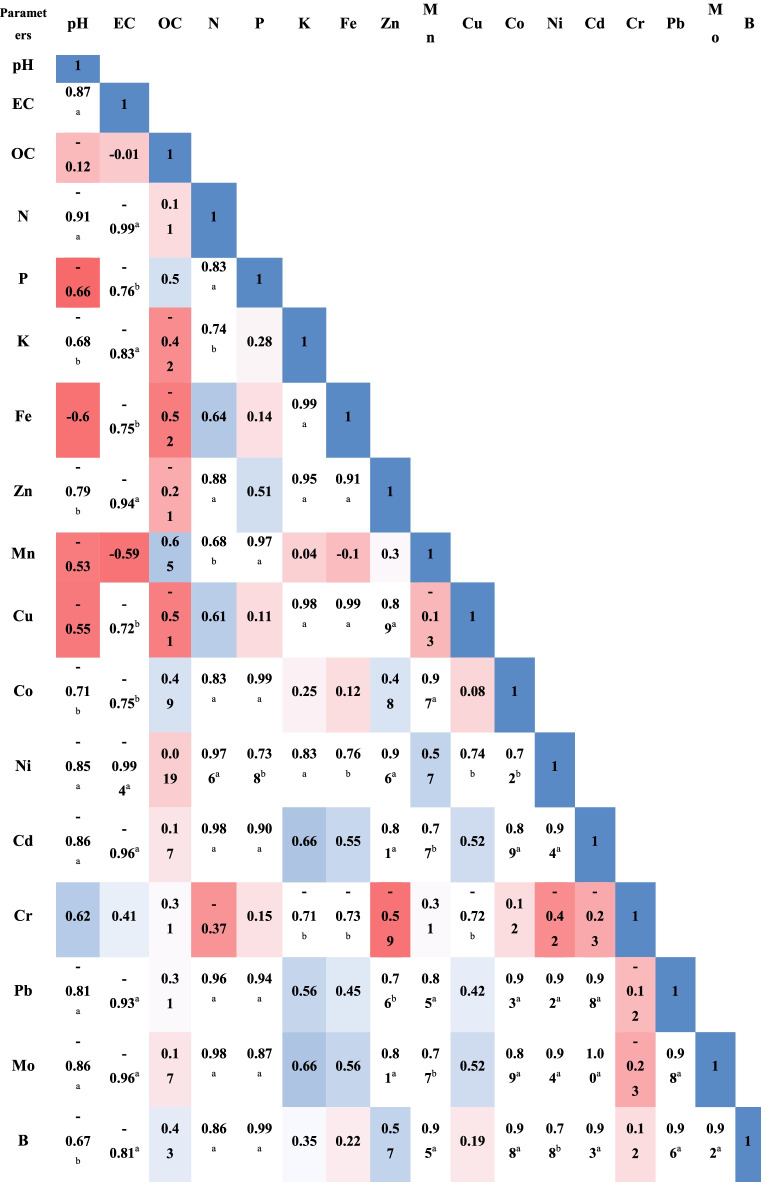
Pearson correlation between geochemical parameters of the study area

^a^Correlation is significant at the 0.01 level

^b^Correlation is significant at the 0.05 level

### Metal concentrations and NPK

N, P, and K contents were significantly higher in plants obtained from site A1 compared to A2 and A3. *A. maurorum* roots at A1 accumulated trace metals as Fe > Ni > Cu > Al > Zn > Mn > Pb > Cr > Cd > Co > B. Similarly, shoots accumulated metals as Fe > Ni > Al > Cu > Zn > Mn > Pb > Cr > Co > Cd (Table 3).

**Table 3:** Heavy metal and macro-nutrient concentrations in different tissues of *Alhagi maurorum* collected from the study area

Element/ Tissue		A1	A2	A3	*F*- value
**Micro-nutrients (Heavy metals) concentrations (mg/kg)**					
**Fe**	**Root**	**225.45 ± 11.524**^**c**^	**8.90 ± 0.208**^**a**^	**137.60 ± 7.843**^**b**^	**183.087**
	**Shoot**	**213.75 ± 11.841**^**c**^	**7.18 ± 0.310**^**a**^	**77.60 ± 4.322**^**b**^	**208.079**
**Zn**	**Root**	**16.15 ± 0.566**^**a**^	**19.73 ± 0.935**^**b**^	**15.32 ± 0.750**^**a**^	**9.376**
	**Shoot**	**18.15 ± 0.589**^**ab**^	**19.31 ± 0.693**^**b**^	**15.43 ± 0.704**^**a**^	**8.995**
**Mn**	**Root**	**101.23 ± 5.324**^**b**^	**161.80 ± 84.686**^**a**^	**18.22 ± 0.808**^**a**^	**174.263**
	**Shoot**	**195.70 ± 10.883**^**a**^	**190.30 ± 9.925**^**c**^	**18.54 ± 0.885**^**a**^	**3.638**
**Ni**	**Root**	**14.93 ± 0.530**^**a**^	**15.32 ± 0.462**^**a**^	**15.20 ± 0.872**^**a**^	**0.352**
	**Shoot**	**15.43 ± 0.819**^**a**^	**15.43 ± 0.398**^**a**^	**15.56 ± 0.606**^**a**^	**0.003**
**Cr**	**Root**	**12.30 ± 0.398**^**b**^	**6.53 ± 0.242**^**a**^	**5.16 ± 0.103**^**a**^	**100.914**
	**Shoot**	**16.62 ± 0.774**^**c**^	**13.09 ± 0.930**^**b**^	**7.70 ± 0.243**^**a**^	**70.143**
**Pb**	**Root**	**101.50 ± 4.619**^**a**^	**100.40 ± 5.277**^**a**^	**101.00 ± 0.462**^**a**^	**0.015**
	**Shoot**	**102.00 ± 4.619**^**a**^	**101.00 ± 5.600**^**a**^	**101.40 ± 5.687**^**a**^	**0.018**
**B**	**Root**	**0.68 ± 0.014**^**c**^	**0.16 ± 0.005**^**a**^	**0.77 ± 0.009**^**b**^	**2958.499**
	**Shoot**	**0.13 ± 0.004**^**a**^	**0.15 ± 0.001**^**a**^	**0.52 ± 0.017**^**b**^	**442.859**
**Mo**	**Root**	**15.00 ± 0.572**^**b**^	**15.16 ± 0.672**^**b**^	**11.80 ± 0.427**^**a**^	**9.935**
	**Shoot**	**13.40 ± 0.468**^**ab**^	**10.22 ± 0.507**^**a**^	**11.31 ± 0.522**^**a**^	**21.977**
**Cu**	**Root**	**4.59 ± 0.087**^**c**^	**2.50 ± 0.017**^**a**^	**2.12 ± 0.060**^**a**^	**372.091**
	**Shoot**	**12.75 ± 0.710**^**c**^	**3.30 ± 0.035**^**b**^	**5.60 ± 0.237**^**b**^	**147.864**
**Co**	**Root**	**30.00 ± 1.155**^**a**^	**30.93 ± 1.698**^**a**^	**30.56 ± 0.883**^**a**^	**0.053**
	**Shoot**	**30.31 ± 1.345**^**a**^	**30.94 ± 1.703**^**a**^	**31.89 ± 1.651**^**a**^	**0.385**
**Cd**	**Root**	**15.40 ± 0.722**^**a**^	**15.10 ± 0.606**^**a**^	**14.16 ± 0.601**^**a**^	**1.338**
	**Shoot**	**15.56 ± 0.768**^**a**^	**15.50 ± 0.791**^**a**^	**16.69 ± 0.722**^**a**^	**0.669**
**Macro-nutrients (ppm)**					
**N**	**Root**	**18,000.0 ± 980.918**^**c**^	**5000.0 ± 248.838**^**a**^	**6000.0 ± 302.53**^**a**^	**102.481**
	**Shoot**	**25,500.0 ± 1385.06**^**b**^	**21,000.0 ± 1155.28**^**b**^	**9500.0 ± 501.717**^**b**^	**93.509**
**K**	**Root**	**4539.00 ± 228.631**^**c**^	**890.0 ± 28.290**^**a**^	**1068.00 ± 45.611**^**a**^	**110.375**
	**Shoot**	**9612.0 ± 520.193**^**b**^	**3738.00 ± 188.216**^**b**^	**1691.0 ± 45.611**^**a**^	**254.967**
**P**	**Root**	**3676.59 ± 159.112**^**b**^	**720.90 ± 55.945**^**a**^	**865.08 ± 20.738**^**a**^	**77.982**
	**Shoot**	**7785.72 ± 395.323**^**b**^	**3027.80 ± 240.051**^**b**^	**1369.71 ± 45.443**^**a**^	**283.318**

The results are recorded as Mean of triplicates ± Standard Error (SE). Different superscript letters refer to significant differences at (*P* < 0.05) level

### Phytoremediation efficiency of *A. maurorum*

HM accumulation and upward translocation were evaluated using BCFs, a simple method for quantitative characterization of available HM uptake from soil to plant. BCF was > 1 for Fe, Zn, Cd, Co, Cr, and Pb, but < 1 for B, Mn, and Ni at all sites. Further, BCFs were less than unity for Cu at A2 and for Mo at A1 and A2 (Table 4).

**Table 4:** Phytoremediation potential: bioaccumulation factor (BCF), translocation factor (TF), shoot coefficient factor (SCF) and root coefficient factor (RCF) for *A. maurorum* at three studied sites

Element/ parameter		A1	A2	A3	*F*-value
**Fe**	**BCF**	**17.98 ± 1.112**^**b**^	**1.54 ± 0.098**^**a**^	**34.01 ± 0.999**^**c**^	**352.465**
	**TF**	**0.95 ± 0.032**^**b**^	**0.81 ± 0.012**^**b**^	**0.56 ± 0.043**^**a**^	**37.467**
	**SCF**	**8.75 ± 0.687**^**b**^	**0.68 ± 0.049**^**a**^	**12.22 ± 0.260**^**c**^	**193.831**
	**RCF**	**9.22 ± 0.427**^**b**^	**0.85 ± 0.049**^**a**^	**21.79 ± 1.257**^**c**^	**188.881**
**Mn**	**BCF**	**0.24 ± 0.012**^**b**^	**0.07 ± 0.003**^**a**^	**0.19 ± 0.017**^**b**^	**53.725**
	**TF**	**1.93 ± 0.064**^**b**^	**1.18 ± 0.001**^**a**^	**1.02 ± 0.078**^**a**^	**69.680**
	**SCF**	**24.49 ± 1.926**^**c**^	**10.99 ± 0.700**^**b**^	**3.46 ± 0.245**^**a**^	**79.992**
	**RCF**	**12.65 ± 0.583**^**c**^	**9.34 ± 0.595**^**b**^	**3.39 ± 0.020**^**a**^	**95.100**
**Pb**	**BCF**	**6.17 ± 0.124**^**a**^	**4.49 ± 0.202**^**a**^	**101.31 ± 1.831**^**b**^	**2702.955**
	**TF**	**1.01 ± 0.064**^**a**^	**1.01 ± 0.081**^**a**^	**1.01 ± 0.090**^**a**^	**0.001**
	**SCF**	**3.09 ± 0.035**^**a**^	**2.25 ± 0.191**^**a**^	**50.62 ± 1.386**^**b**^	**1173.761**
	**RCF**	**3.08 ± 0.159**^**a**^	**2.23 ± 0.012**^**a**^	**50.69 ± 3.217**^**b**^	**222.388**
**Co**	**BCF**	**7.03 ± 0.306**^**a**^	**3.37 ± 0.116**^**a**^	**34.78 ± 1.531**^**b**^	**361.254**
	**TF**	**1.02 ± 0.087**^**a**^	**1.00 ± 0.002**^**a**^	**1.04 ± 0.078**^**a**^	**0.105**
	**SCF**	**3.54 ± 0.306**^**a**^	**1.69 ± 0.058**^**a**^	**17.80 ± 1.432**^**b**^	**108.503**
	**RCF**	**3.49 ± 0.001**^**b**^	**1.69 ± 0.061**^**a**^	**16.99 ± 0.098**^**c**^	**15,744.450**
**Zn**	**BCF**	**11.21 ± 0.020**^**a**^	**17.01 ± 0.685**^**b**^	**24.43 ± 0.497**^**c**^	**184.221**
	**TF**	**1.12 ± 0.003**^**a**^	**0.99 ± 0.095**^**a**^	**1.01 ± 0.075**^**a**^	**1.052**
	**SCF**	**5.93 ± 0.023**^**a**^	**8.39 ± 0.058**^**b**^	**12.24 ± 0.208**^**c**^	**643.149**
	**RCF**	**5.28 ± 0.003**^**a**^	**8.62 ± 0.745**^**b**^	**12.20 ± 0.705**^**c**^	**34.165**
**Cr**	**BCF**	**4.19 ± 0.061**^**c**^	**2.48 ± 0.026**^**b**^	**1.65 ± 0.084**^**a**^	**443.365**
	**TF**	**1.36 ± 0.104**^**a**^	**2.01 ± 0.188**^**b**^	**1.50 ± 0.121**^**ab**^	**5.799**
	**SCF**	**2.41 ± 0.043**^**c**^	**1.65 ± 0.069**^**b**^	**0.99 ± 0.018**^**a**^	**216.698**
	**RCF**	**1.79 ± 0.104**^**b**^	**0.83 ± 0.043**^**a**^	**0.67 ± 0.064**^**a**^	**65.785**
**Ni**	**BCF**	**0.59 ± 0.009**^**a**^	**0.56 ± 0.017**^**a**^	**4.46 ± 0.127**^**b**^	**912.759**
	**TF**	**1.04 ± 0.081**^**a**^	**1.01 ± 0.049**^**a**^	**1.03 ± 0.116**^**a**^	**0.021**
	**SCF**	**0.30 ± 0.006**^**a**^	**0.28 ± 0.001**^**a**^	**2.24 ± 0.064**^**b**^	**935.757**
	**RCF**	**0.29 ± 0.017**^**a**^	**0.28 ± 0.015**^**a**^	**2.21 ± 0.191**^**b**^	**101.071**
**Cd**	**BCF**	**3.39 ± 0.116**^**a**^	**2.81 ± 0.032**^**a**^	**27.56 ± 0.390**^**b**^	**3599.182**
	**TF**	**1.03 ± 0.081**^**a**^	**1.03 ± 0.020**^**a**^	**1.19 ± 0.121**^**a**^	**1.280**
	**SCF**	**1.72 ± 0.121**^**a**^	**1.42 ± 0.032**^**a**^	**14.93 ± 0.904**^**b**^	**214.517**
	**RCF**	**1.67 ± 0.012**^**a**^	**1.39 ± 0.001**^**a**^	**12.62 ± 0.511**^**b**^	**471.226**
**Mo**	**BCF**	**0.95 ± 0.006**^**a**^	**0.74 ± 0.049**^**a**^	**3.30 ± 0.095**^**b**^	**525.331**
	**TF**	**0.89 ± 0.009**^**b**^	**0.67 ± 0.006**^**a**^	**0.96 ± 0.012**^**c**^	**283.136**
	**SCF**	**0.45 ± 0.001**^**b**^	**0.30 ± 0.023**^**a**^	**1.62 ± 0.038**^**c**^	**801.571**
	**RCF**	**0.50 ± 0.006**^**a**^	**0.44 ± 0.029**^**a**^	**1.68 ± 0.058**^**b**^	**350.235**
**Cu**	**BCF**	**1.01 ± 0.020**^**a**^	**0.91 ± 0.029**^**a**^	**2.02 ± 0.121**^**b**^	**70.260**
	**TF**	**2.79 ± 0.159**^**b**^	**1.33 ± 0.069**^**a**^	**2.65 ± 0.156**^**b**^	**35.914**
	**SCF**	**0.75 ± 0.003**^**a**^	**0.52 ± 0.006**^**a**^	**1.47 ± 0.113**^**b**^	**57.950**
	**RCF**	**0.27 ± 0.017**^**a**^	**0.39 ± 0.026**^**b**^	**0.55 ± 0.009**^**c**^	**57.358**
**B**	**BCF**	**0.32 ± 0.020**^**b**^	**0.09 ± 0.003**^**a**^	**0.92 ± 0.012**^**c**^	**995.820**
	**TF**	**0.19 ± 0.002**^**a**^	**0.94 ± 0.034**^**c**^	**0.68 ± 0.003**^**b**^	**358.633**
	**SCF**	**0.05 ± 0.003**^**a**^	**0.04 ± 0.001**^**a**^	**0.37 ± 0.006**^**b**^	**2355.250**
	**RCF**	**0.27 ± 0.017**^**b**^	**0.04 ± 0.003**^**a**^	**0.55 ± 0.006**^**c**^	**561.032**

The results are recorded as Mean of triplicates ± Standard Error (SE). Different superscript letters refer to significant differences at (*P* < 0.05) level

The TF, a measure of metal transport from roots to shoots, was greater than one for Mn, Pb, Co, Zn, Cr, Ni, Cd, and Cu, and less than unity for Fe, Mo, and B, at all sites.

HM accumulation was determined by SCF and exceeded unity at all sites for Zn, Mn, Cd, Pb, and Co, but was < 1 for B. SCF was less than unity for Cu, Ni, and Mo at A1 and A2; for Fe at A2; for Cr at A3 (Table 4).

RCF exceeded unity for Mn, Pb, Cd, Co, and Zn at all sites and was > 1 for Fe at A1 and A3, for Mo and Ni at A3, and for Cr at A1. The RCF for Cu and B was less than unity at all sites (Table 4).

### Biochemical parameters of *A. maurorum* shoots

Analysis for bioorganic substances can detect important macromolecules, including pigments and primary and secondary metabolites, which could regulate various biological processes.

### Non-enzymatic antioxidant activities

**Fig. 1:**
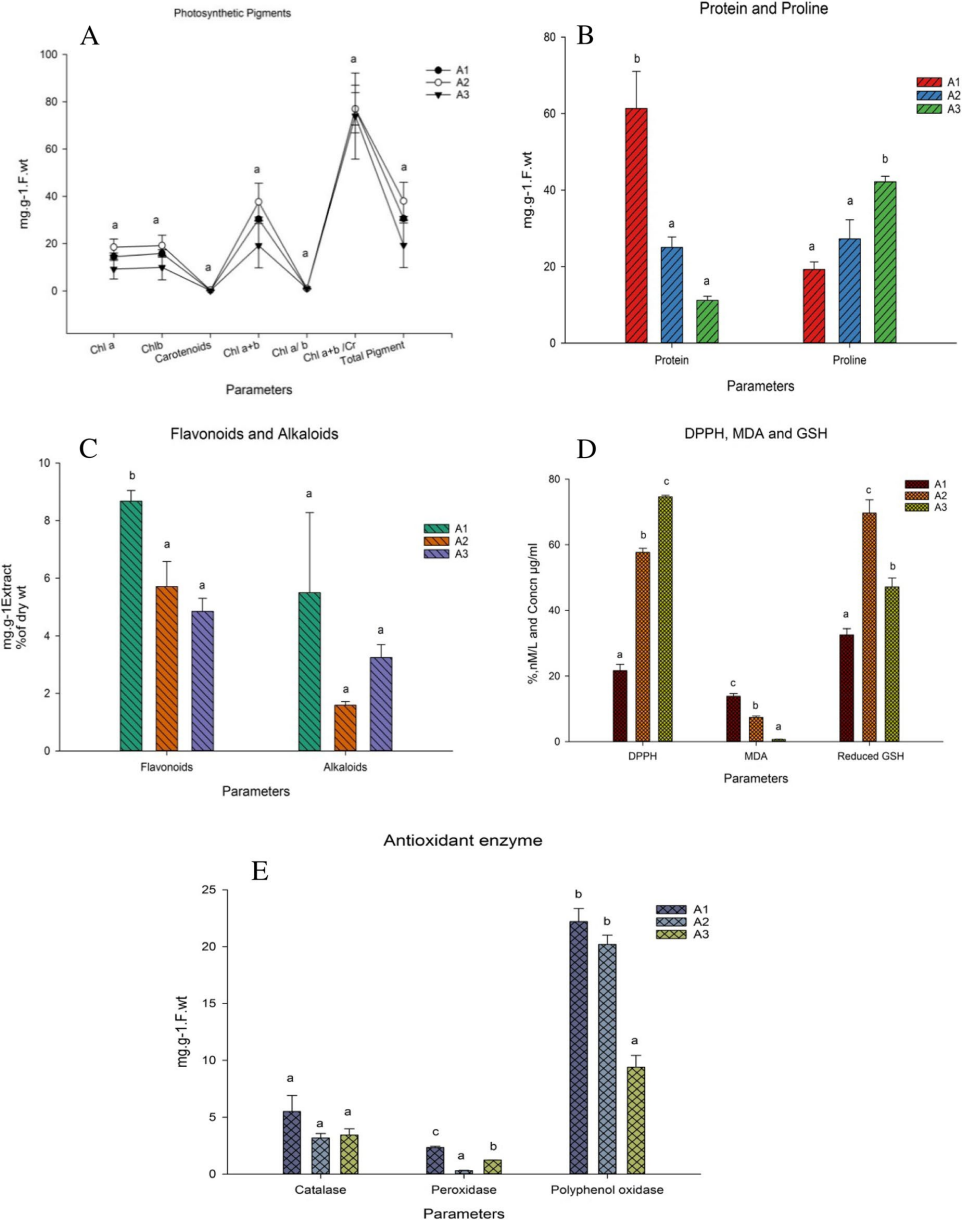
Quantification of biochemical parameters of *A. maurorum* shoots collected from the studied sites A1, A2 and A3. **A** Photosynthetic pigments (chlorophyll a, chlorophyll b, carotenoids, Chl a + Chl b, Chl a/Chl b, Chl a + Chl b/Carotenoids, and total pigments); **B** protein and proline; **C** flavonoids and alkaloids; **D** DPPH, malondialdehyde (MDA) and reduced glutathione (GSH); **E** antioxidant enzymes (catalase, peroxidase and polyphenol oxidase)

Chlorophyll a levels in leaves were 18.49 (A2) > 14.50 (A1) > 9.24 (A3) mg. g^− 1^ fresh wt. Chlorophyll b levels were 19.16 (A2) > 15.84 (A1) **>** 9.92 (A3) mg. g^− 1^ fresh wt. Carotenoids exhibited concentrations 0.27, 0.33, and 0.14 mg. g^− 1^ fresh wt. for A1, A2, and A3, respectively. Moreover, total pigments were calculated as 30.61, 37.97, and 19.30 mg. g^− 1^ fresh wt, for A1, A2, and A3, respectively (Fig. 1A). Significant differences in protein content of *A. maurorum* shoots were 61.38 mg. g^− 1^ fresh wt. at A1 and 11.19 mg. g^− 1^ fresh wt. at A3. Proline content was highest at A3, 42.21 mg. g^− 1^ fresh wt. (Fig. 1B).

Total flavonoid content gradually decreased as 8.68 (A1) > 5.71 (A2) > 4.84 (A3) mg. g^**− 1**^ dry wt. Alkaloid content of shoot differed as 5.50 (1A) > 3.25 (A3) > 1.59 (A2) % dry weight (Fig. 1C).

**Fig. 2:**
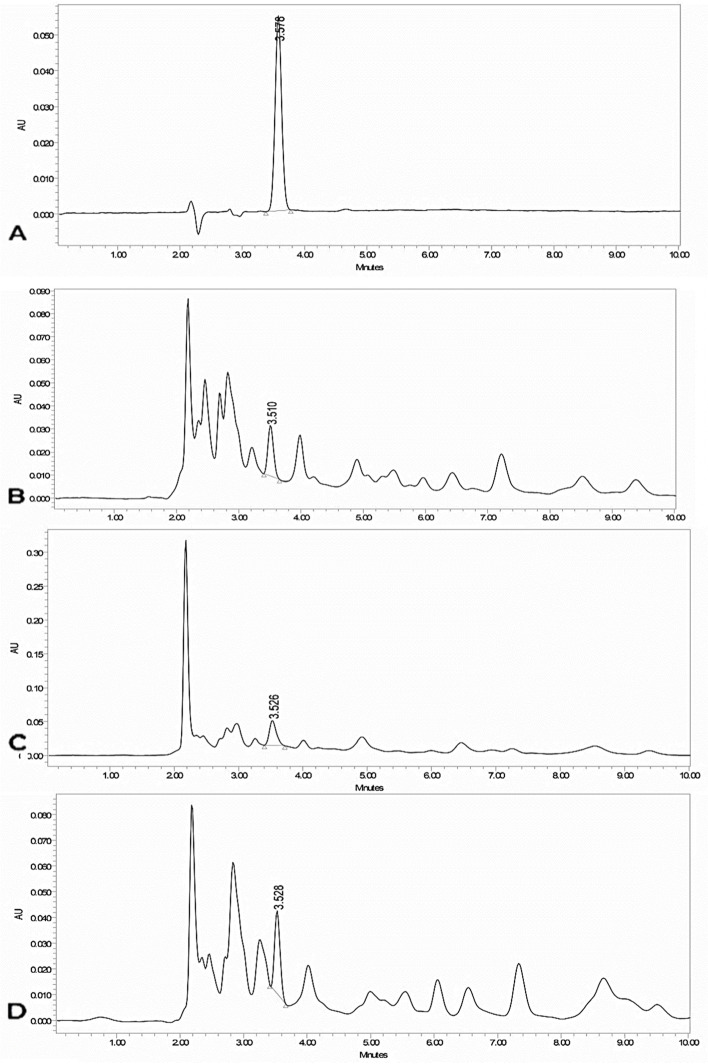
Chromatogram of HPLC for reduced glutathione assay of *Alhagi maurorum* samples*.* **A** Standard (100 μg/mL); **B** A1; **C** A2; **D** A3

Shoot extracts showed significant DPPH free radical scavenging impacts compared to ascorbic acid. The DPPH scavenging varied as 21.63, 57.68, and 74.63% at A1, A2, and A3, respectively (Fig. 1D). Lipid peroxidation levels in shoots gradually decreased as 13.85 at A1, 7.41 at A2 and 0.71 nM/L MDA at A3 (Fig. 1D). Reduced GSH analytes appeared as major peaks at retention times of 2.62 and 3.50 min. The HPLC chromatogram suggested that reduced GSH was detected under HM stress at 3.50 min (Fig. 2). GSH concentrations in leaves from A1, A2, and A3 were 32.59, 69.67, and 47.14 μg/mL, with retention times of 3.51, 3.53, and 3.53 min, respectively (Table 5 and Fig. 1D).

**Table 5:** Concentration, area and retention time of reduced glutathione of standard of *A. maurorum* samples

Sample code	Concentration (μg/mL)	Rt (min)	Area
**GSH** **Standard**	50	3.562	206,365
	100	3.578	398,534
	150	3.581	564,721
	200	3.578	758,831
	250	3.596	966,297
**A1**	32.59128	3.510	137,455
**A2**	69.67321	3.526	276,895
**A3**	47.14322	3.528	192,175

### Enzymatic antioxidant stress modulators

Antioxidant enzyme activities for *A. maurorum* shoots were highest at site A1 for CAT (5.50 mg·g^− 1^ fresh wt.), POD (2.34 mg·g^− 1^ fresh wt.) and polyphenol oxidase (PPO) (22.20 mg·g^− 1^ fresh wt.) activities (Fig. 1E).

### Semi-thin ultrastructure of *A. maurorum* shoots

#### Description of leaf sections

**Fig. 3:**
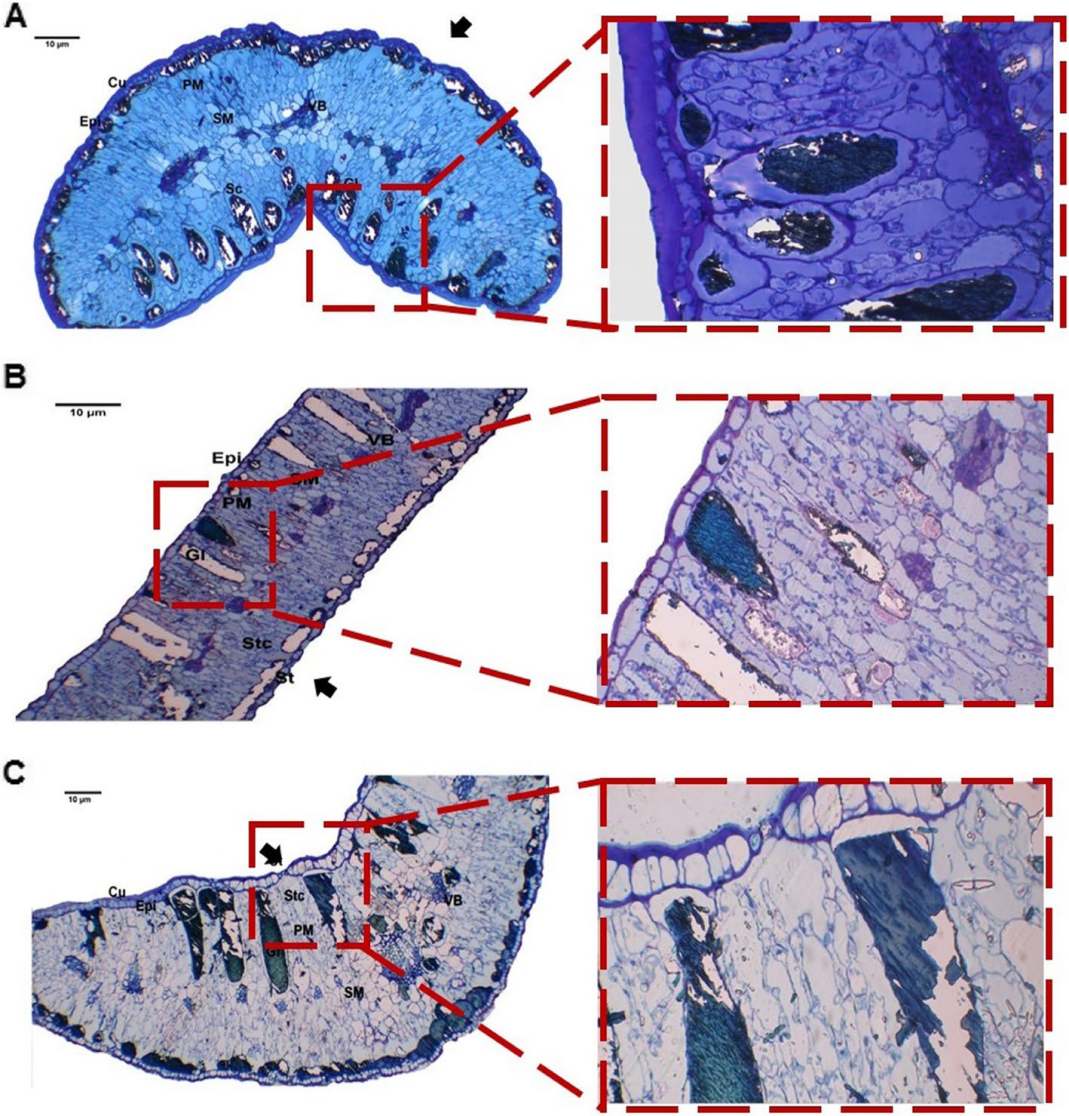
Histological analysis of transverse section of *A. maurorum* leaf phenotype at 100X and magnified parts at 400X. **A** Leaf intercostal region and margin of site A1 and its magnified part showing gland of site A1; **B** leaf midrib of site A2 and its magnified part showing gland of site A2; **C** Leaf intercostal region and margin of site A3 and its magnified part showing stomata. Black arrows indicated stomata openings; Figure abbreviations: Cu, cuticle; Epi, epidermis; PM, palisade mesophyll; SM, spongy mesophyll; VB, vascular bundle; Xy, xylem; Ph, phloem; SC, secretory cavity; Gl, gland; St, stomata; Stc, stomatal cavity. Bars = 10 μm

Histological analysis of transverse sections of leaf blades showed typical three tissue differentiation: epidermis, mesophyll, and vascular. The outermost layer consisted of adaxial and abaxial thick-walled elongated epidermal cells covered with a thick layer of cuticle. Tanninferous cells or glands were distributed as a compact layer of small cells underneath the lower epidermis or large scattered cells in the periphery in the upper palisade parenchyma. Mesophyll tissue was bifacial and differentiated into palisade and spongy. The leaf was dorsiventral, and palisade mesophyll parenchyma was found on both adaxial and abaxial sides with a strip of spongy parenchyma in the middle portion of the lamina. Vascular bundles of the midvein were open collateral with narrow cambial tissue (Fig. 3).

**Fig. 4:**
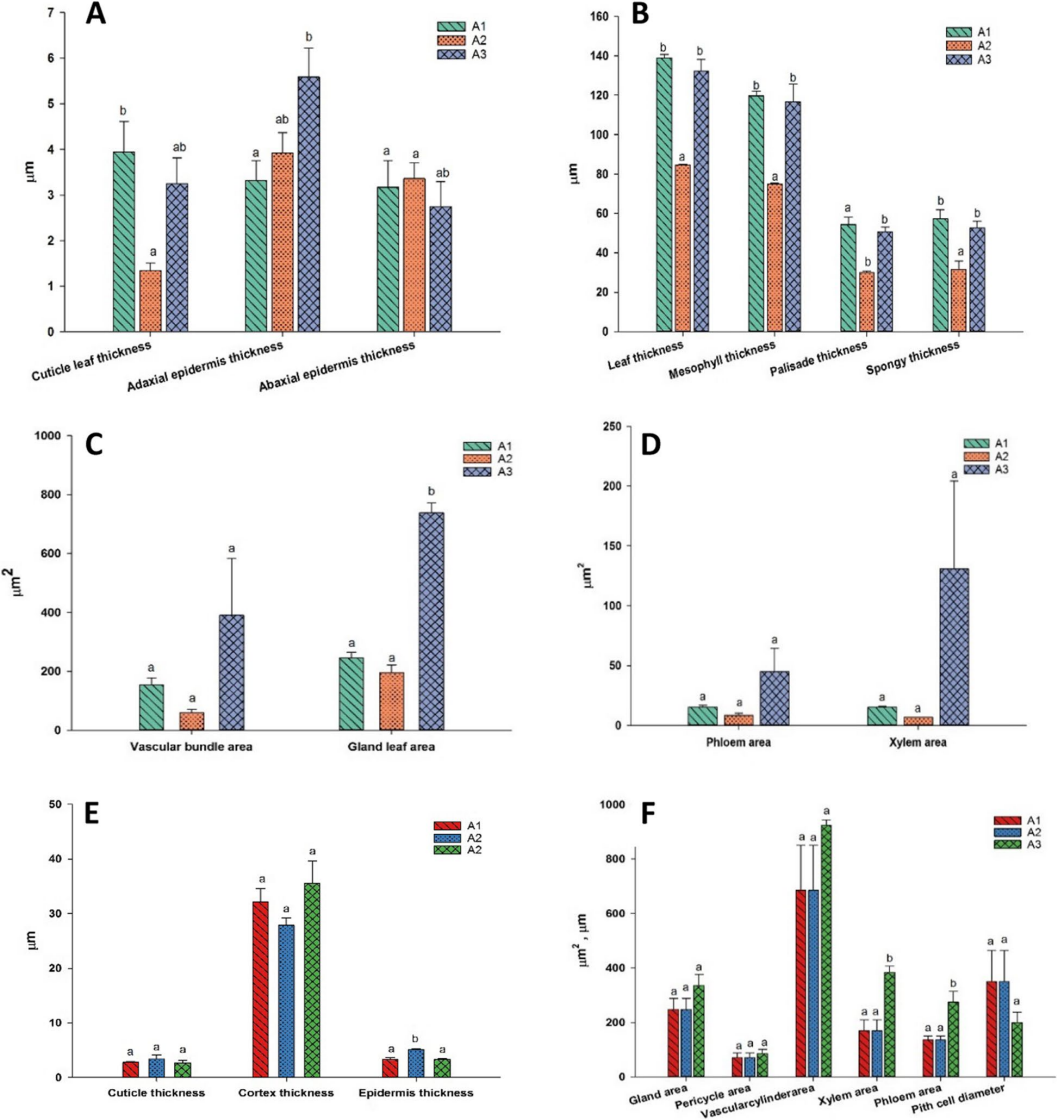
Phenotypic measurements of leaf and stem semi-thin sections of *A. maurorum* collected from the studied area A1, A2 and A3. **A**-**D** showed the leaf anatomical parameters while, **E**-**F** showed stem anatomical changes among A1, A2 and A3. One-way ANOVA results presented as Means ± Standard error superscripted by the same letter in the histogram do not differ (Tukey Post Hoc test, *p* ≤ 0.05, *n* = 3)

The greatest leaf thickness was recorded at site A1 and the lowest at A2. The cuticular thickness of the adaxial epidermal surface was more than the abaxial surface (Fig. 4A–D). Moreover, tanninferous cells or tannin glands accumulated underneath the lower epidermis as small cells and scattered as large cells under the upper epidermis. The number and area of the tanninferous cells decreased as A3 > A1 > A2 sites. The palisade mesophyll consisted of 3–4 layers of elongated parenchyma with abundant chloroplasts and tanninferous cells toward the adaxial epidermis. Mesophyll with palisade and spongy thickness was maximum at site A1 > A3 and A2. Vascular bundle area was highest at site A3 and lowest at A2. *A. maurorum* collected from A3 site showed the widest metaxylem and thickest phloem.

#### Description of stem sections

The results of the anatomical features of the *A. maurorum* stem between the three studied sites were detected in Fig. 4 (E and F). The epidermal cells were covered by a thick layer of cuticle, except for the openings of the sunken stomata pores. The highest epidermal cell thickness was scored in A2 > A1 > A3 sites. The *A. maurorum* stem was considered a xerophytic type, with a maximum chlorenchyma thickness for A3 site and minimum for A2 site. Maximum vascular bundle thickness was recorded for A3 site, while the minimum was for A1. Furthermore, *A. maurorum* present at A3 site had thicker and stronger xylem and phloem fibres. Pith parenchymatous cell diameter was the widest in *A. maurorum* of A1, A2 and A3 sites.

**Fig. 5:**
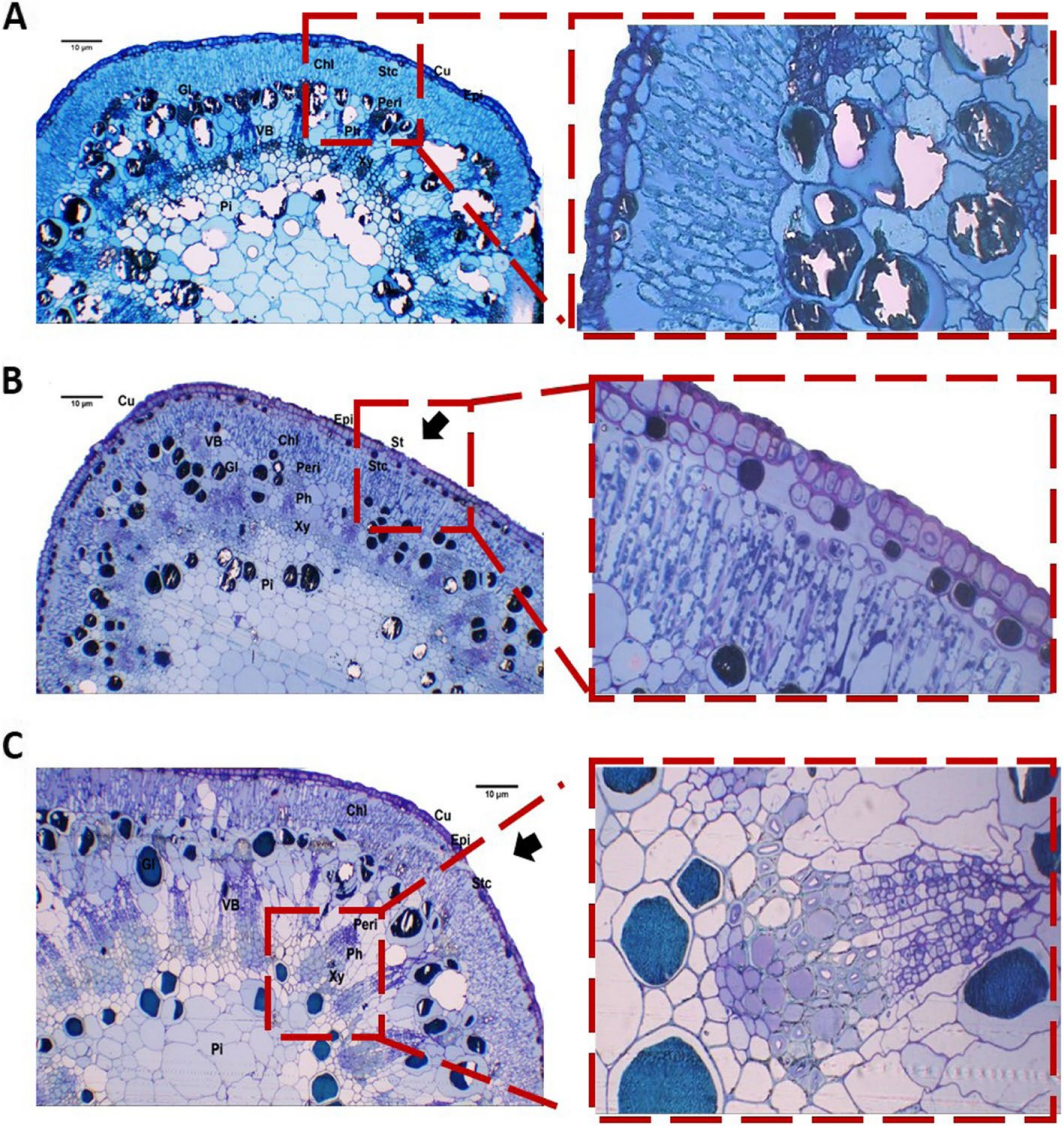
Histological analysis of transverse section of *A. maurorum* stem showing stem phenotypic properties at 100X and magnified parts at 400X. **A** Stem of site A1 and its magnified part showing chlorenchyma with glands; **B** Stem of site A2 and its magnified part showing stomata and stomatal cavity; **C** Stem site of A3 and its magnified part showing vascular bundle. Black arrows indicated stomata openings; Figure abbreviations: Cu, Cuticle; Epi, epidermis; Stc, stomatal cavity; Chl, chlorenchyma; Gl, gland; Peri, pericycle; VB, vascular bundle; Xy, xylem; Ph, phloem; Pi, pith. Bars = 10 μm

Transverse stem sections of *A. maurorum* stem (Fig. 5) also showed three tissue types: epidermal, ground, and vascular bundles. The epidermis consisted of compact elongated parenchymal cells with several sunken stomata. One compact single layer was found underneath the epidermis, called the hypodermis, with several small tanninferous cells containing tannins. Ground tissue differentiated into cortex and pith. The former consisted of several layers of chlorenchyma, with thin-walled parenchyma beneath these layers. These layers consisted of parenchymatous cells occupying the central core of the stem. Stem vascular bundles were differentiated into small and large open collateral types. Medullary rays were located between large phloem fibers. Tanninferous cells in the *A. maurorum* stem were scattered in the cortex, pith, and medullary rays.

### Intercorrelation between heavy metals and different responses of *A. maurorum*

**Fig. 6:**
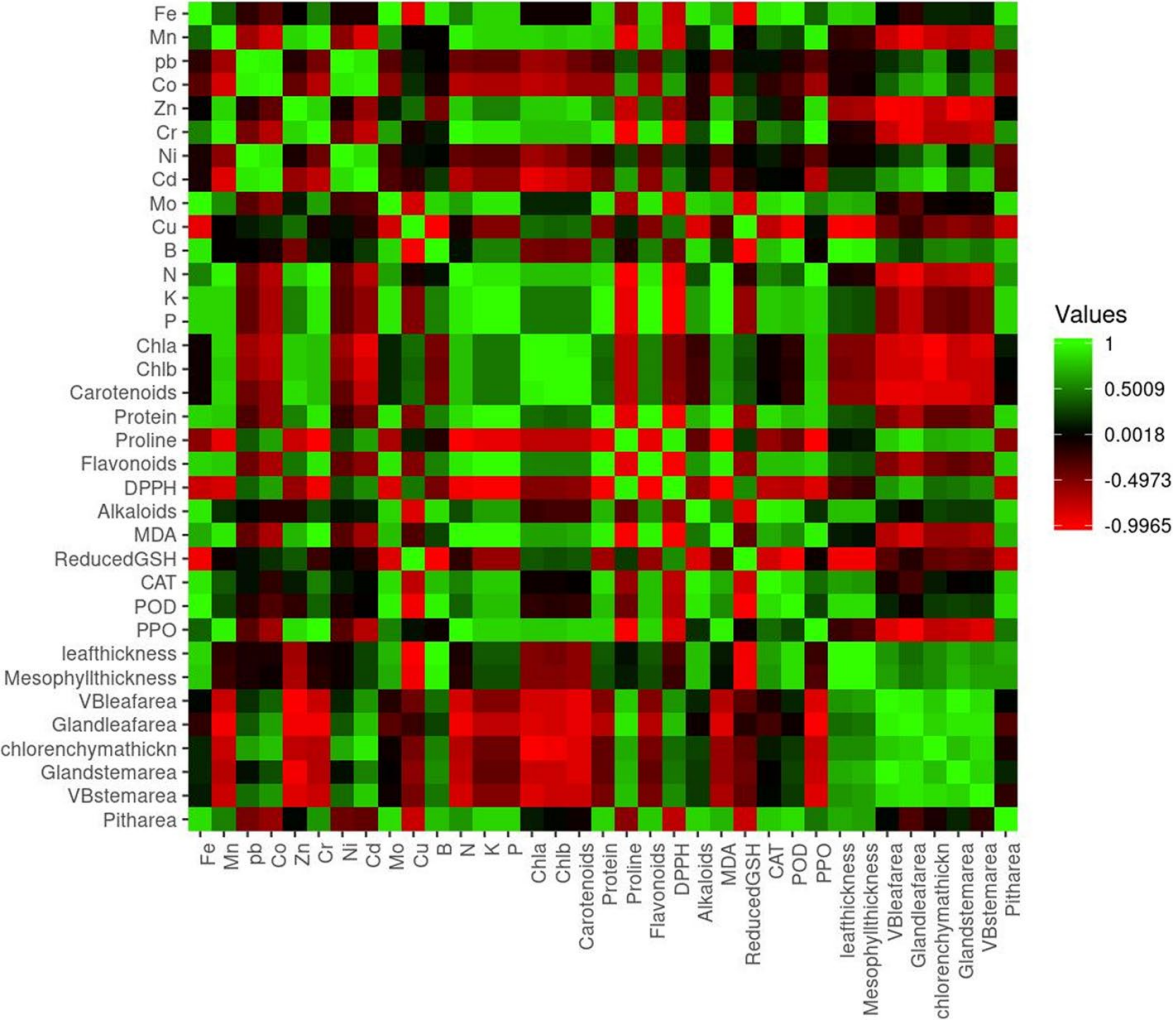
Heatmap of simple linear Pearson correlation coefficients between heavy metals, biochemical parameters and anatomical features of *A. maurorum* shoot system*.* Correlation levels are colored green for high intensities and red for low intensities (follow scale at the top right corner)

The heatmap provides classification of all the studied biochemical and anatomical parameters of the *A. maurorum* shoot system using Pearson correlation (Fig. 6). Fe had significantly strong positive correlations with other HMs such as Mo and Cu, as well as with K, P, POD, protein, flavonoids and mesophyll thickness. On the other hand, Fe was negatively correlated with DPPH, reduced GSH and gland leaf area. Mn was positively correlated with the other metals (Zn, Cr and Cu). Similarly, Mn was positively correlated with PPO, MDA and pith area, but negatively correlated with Cd, B, proline, gland leaf area and leaf vascular bundle area. Co, Ni and Cd were positively correlated with Pb, but negatively correlated with Mo and photosynthetic pigments. Zn had a positive correlation with all other HMs except Cd, Cu and B. In addition, Zn was negatively correlated with proline, DPPH, alkaloids and POD. Moreover, Mo was positively correlated with Cu, K, P, protein, flavonoids, POD and mesophyll thickness.

Micronutrients (N, P and K) were positively correlated to each other and with Cr and Mn. They also had a positive impact on various parameters such as protein, flavonoids, MDA, POD and PPO. Conversely, changes in N, P and K were inversely correlated with changes in proline, DPPH, reduced GSH, shoot vascular bundle area and chlorenchyma thickness. Additionally, photosynthetic pigments (chlorophyll a, chlorophyll b and carotenoids) showed a positive relationship with each other and with other parameters such as protein, flavonoids, MDA, GSH and PPO, while they were inversely correlated with proline, DPPH, alkaloids, CAT, POD, leaf thickness, mesophyll and chlorenchyma thickness, shoot vascular bundle area and stem gland area. Flavonoids were significantly positively correlated with changes in total protein content. Cu and Fe were closely related to them. Flavonoids and protein content were positively correlated with MDA and enzymatic antioxidants. Additionally, proline was significant closely correlated with DPPH, while it had a negative correlation with protein, flavonoids and MDA. Furthermore, alkaloids were positively correlated with CAT (Fig. 6).

### Molecular genetic analyses

#### Expression analysis of stress-related genes

**Fig. 7:**
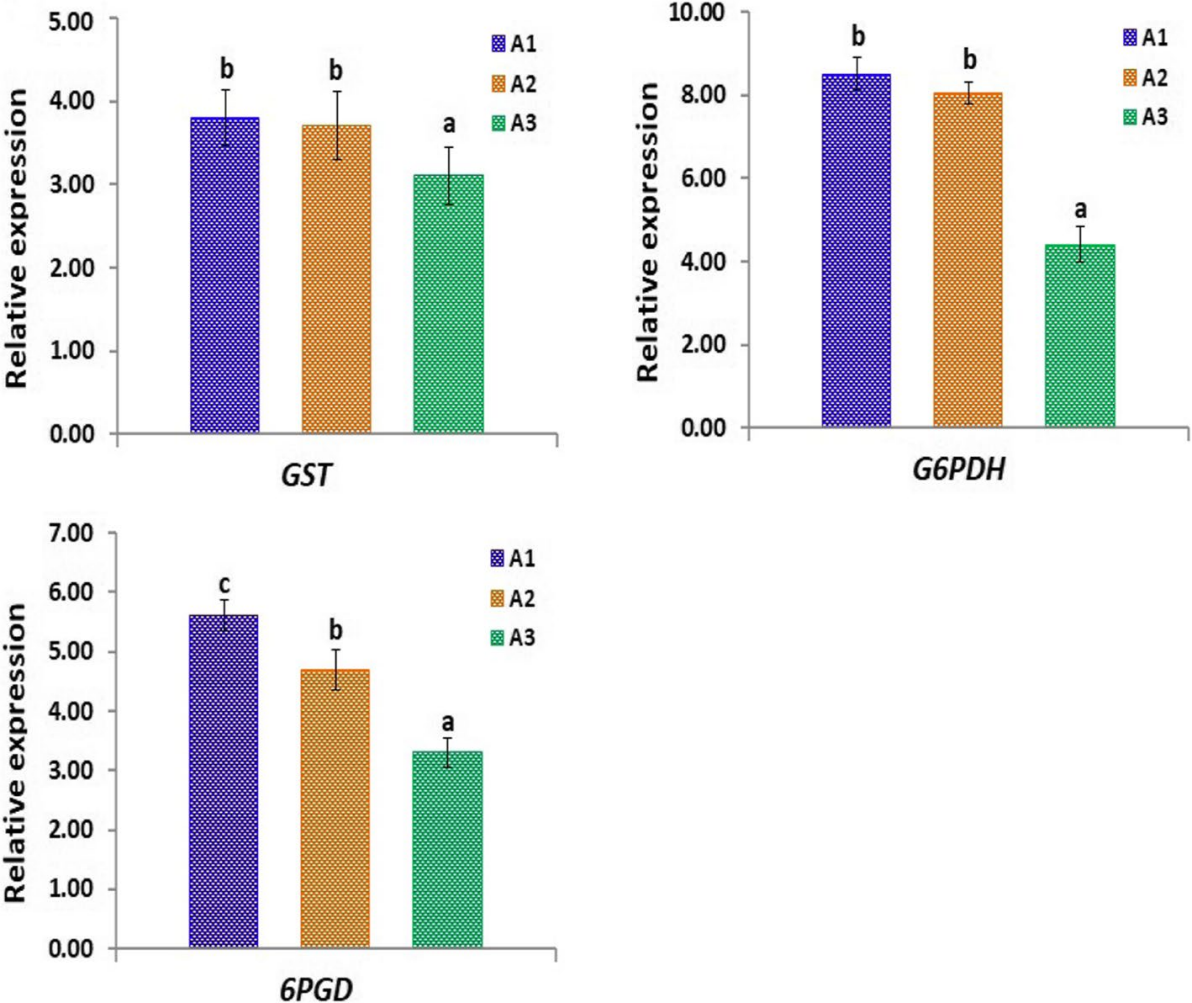
Quantitative RT-PCR of *GST, G6PDH* and *6PGD* genes in *A. maurorum* plants collected from the studied sites A1, A2 and A3

**Fig. 8:**
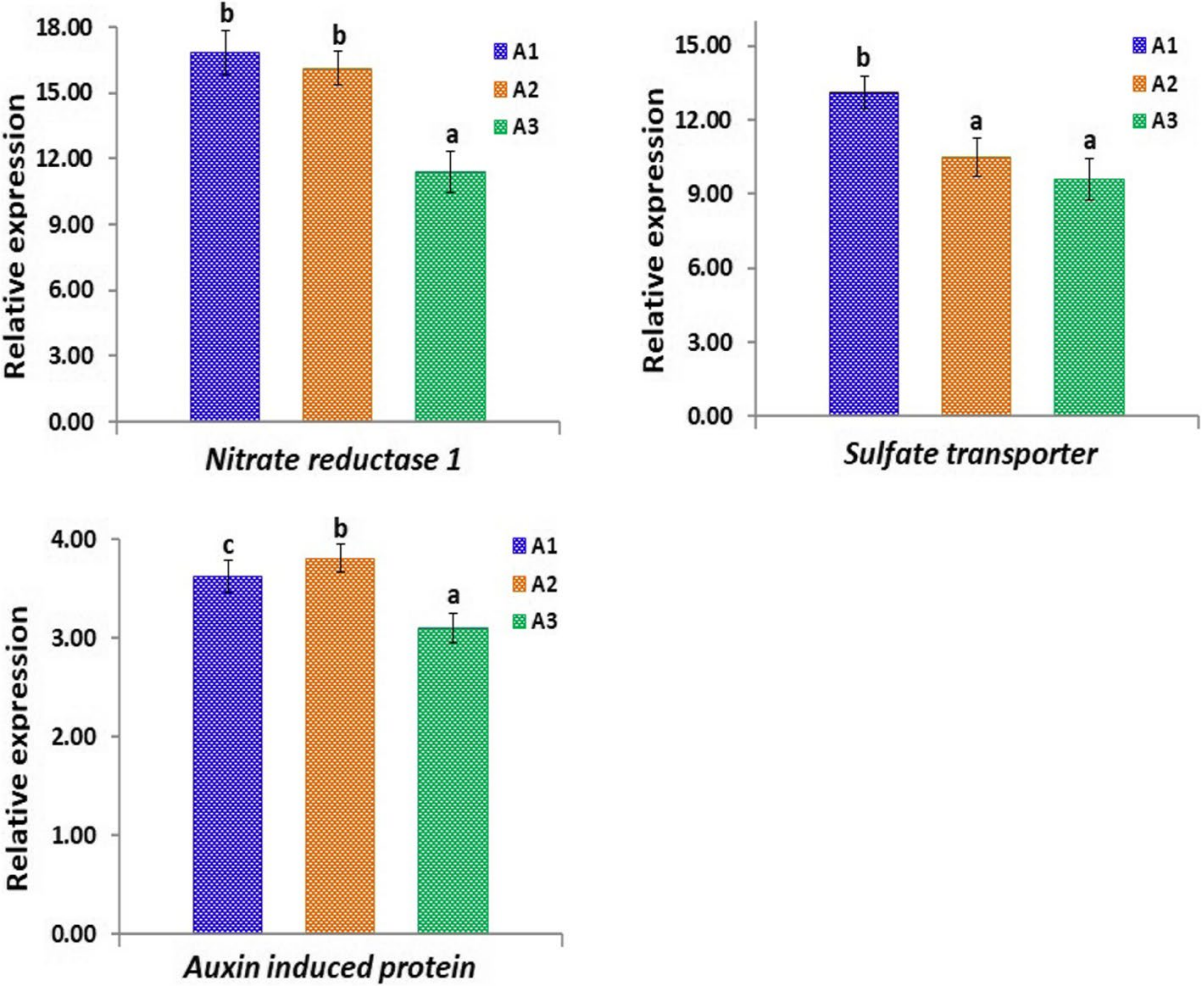
Quantitative RT-PCR of nitrate *reductase 1, sulfate transporter* and *auxin-induced protein* in *A. maurorum* plants collected from the studied sites A1, A2 and A3

In order to investigate the effect of heavy metal accumulation in the three studied sites at the molecular level, the expression levels of 6 genes (*GST, G6PDH, 6PGD, nitrate reductase 1, sulfate transporter* and *auxin-induced protein*) in *A. maurorum* plants collected from the different studied sites were estimated and shown in Figs. 7 and 8. Notably, the highest levels of expression of *GST, G6PDH* and *6PGD* genes have been detected in plants collected from site A1, followed by that recorded in plants collected from A2 and A3 sites (Fig. 7).

Similarly, Fig. 8 shows considerable variations in the expression levels of *nitrate reductase 1, sulfate transporter* and *auxin-induced protein* in *A. maurorum* plants collected from different sites. The highest levels of expression of *nitrate reductase 1* and *sulfate transporter* have been detected in plants collected from site A1. However, *auxin-induced protein* exhibited its highest expression level in plants collected from site A2 (5 and 23% increases over that recorded in plants collected from A1 and A3 sites, respectively). These results indicate the heavy metal accumulation impacts on the expression of stress-related genes in plants collected from the studied sites.

#### Molecular genetic diversity analysis using ISSR and SRAP markers

Five ISSR primers were used and yielded 11 scoreable and reproducible fragments, 7 of which were monomorphic and 4 were polymorphic, with an average polymorphism percentage of 36.36%. MBF values varied from 0.83 in 49A, HB-10 and HB-11 primers to 1.00 in HB-9 primer. As shown in Table 6, each primer yielded distinct amplification products with sizes varying from 200 to 900 bp. The polymorphism percentage for each primer ranged from 33.33% in HB-13 to 50% in 49A, HB-10, and HB-11. Levels of polymorphism and informativeness of ISSR markers were also expressed in some variables such as PIC, DI, and RP. The highest PIC value (0.22) was found in primers (49A, HB-10 and HB-11), while the lowest (0.00) was recorded in primer HB-9. The primers 49A, HB-10, HB-11, and HB-13 showed the highest Rp values (0.67), with an average of 0.53. All ISSR primers had a DI of 0.16 (Table 6).

**Table 6:** Primer sequence, band length, total amplified bands, monomorphic, polymorphic, unique bands and percentage of polymorphism and efficiency of ISSR and SRAP analysis of three *A. maurorum* genotypes

Primer Name	Primer sequence (5`----3`)	Band length (bp)	TAB	NMB	NPB	NUB	PPB	MBF	PIC	RP
**ISSR markers**										
**49A**	CAC ACA CAC ACA AG	215–385	2	1	1	–	50%	0.83	0.22	0.67
**HB-9**	CAC CAC CAC GC	380–435	2	2	–	–	–	1.00	0.00	0.00
**HB-10**	GAG AGA GAG AGA CC	280–470	2	1	1	–	50%	0.83	0.22	0.67
**HB-11**	GTG TGT GTG TGT TGT CC	460–810	2	1	1	–	50%	0.83	0.22	0.67
**HB-13**	GAG GAG GAG C	470–975	3	2	1	–	33.33%	0.89	0.15	0.67
**Total**		–	11	7	4	–	–	–	–	–
**Average**		200–900	2.2	1.4	0.8	–	36.36%	0.88	–	0.53
**DI of all ISSR primers =** 0.16										
**SRAP markers**										
**ME1xEM7**	F ME-1: GAGTCCAAACCGGATA R EM-7: GACTGCGTACGAATTCAA	275–1500	6	–	6	3	100%	0.50	0.45	4.02
**ME1xEM8**	ME-1: GAGTCCAAACCGGATA EM-8: CTGCGTACGAATTCAC	320–680	4	2	2	–	50%	0.83	0.22	1.36
**ME2xEM6**	ME-2: TGA GTC CAA ACC GGA GC EM-6: GAC TGC GTA CGA ATT CC	145–845	7	2	5	2	71.42%	0.66	0.32	3.36
**ME2xEM7**	ME-2: TGA GTC CAA ACC GGA GC Em-7: GACTGCGTACGAATTCAA	135–185	3	2	1	1	33.33%	0.78	0.15	0.66
**ME5xEM6**	Me-5: TGAGTCCAAACCGGAAG EM-6: GAC TGC GTA CGA ATT CC	110–200	3	1	2	1	66.66%	0.66	0.30	1.34
**ME5xEM8**	Me-5: TGAGTCCAAACCGGAAG EM-8: CTGCGTACGAATTCAC	100–250	2	2	–	–	–	1.00	0	0
**Total**		–	25	9	16	7	–	–	–	–
**Average**		100–1500	4.17	1.5	2.67	1.17	64%	0.74	–	1.79
**DI of all SRAP primers** = 0.24										
**Combined ISSR and SRAP**			36	16	20	7	55.55%	–	–	–

*TAB* Total amplified bands, *NMB* Number of monomorphic bands, *NPB* Number of polymorphic bands; *NUB* Number of unique bands, *PPB* Percentage of polymorphic bands, *MBF* Mean of band frequency, *PIC* polymorphism information content, *Rp* Resolving power and *DI* Diversity index

SRAP analysis was also carried out to validate ISSR analysis results. Out of the 6 SRAP primers combinations screened, 25 amplicons in total were generated with 9 monomorphic and 16 polymorphic. MBF values in ME1xEM7 primer combinations and ME1xEM8 primer combinations varied from 0.5 to 0.83, respectively. The scorable fragments ranged in size from 100 to 1500 bp with an average polymorphism rate of 64%. ME1xEM7 primer showed the highest polymorphism (100%) followed by 71.42% in ME2xEM6, 66.66% in ME5xEM6, 50% in ME1xEM8 and lasted with 33.33% in ME2xEM7, with no percentage in ME5xEM8 (Table 6). Three out of the 7 unique bands were found in ME1xEM7 at the molecular sizes of 260, 490 and 1500 bp in *A. maurorum* at A1 site. ME2xEM6 primer has 2 unique bands of molecular sizes of 300 bp in *A. maurorum* at site A3 and 845 bp in *A. maurorum* at A1 site. Likewise, ME2xEM7 primer has one unique band of 190 bp in *A. maurorum* at A1 site and ME5xEM6 primer has a 140 bp unique band in *A. maurorum* at A1 site. Some parameters such as PIC, DI and RP indicated the ISSR markers effectiveness. ME1xEM7 primers showed the highest PIC (0.45), whereas ME5xEM8 primers had the lowest (0.00). The highest Rp value of the ME1xEM7 primers combination was 4.02, followed by 3.36 for ME2xEM6, and the lowest Rp value was 0.00 for the ME5xEM8 primers combination, with an average of 1.79. The DI of all SRAP primer combinations was 0.24 (Table 6).

**Fig. 9:**
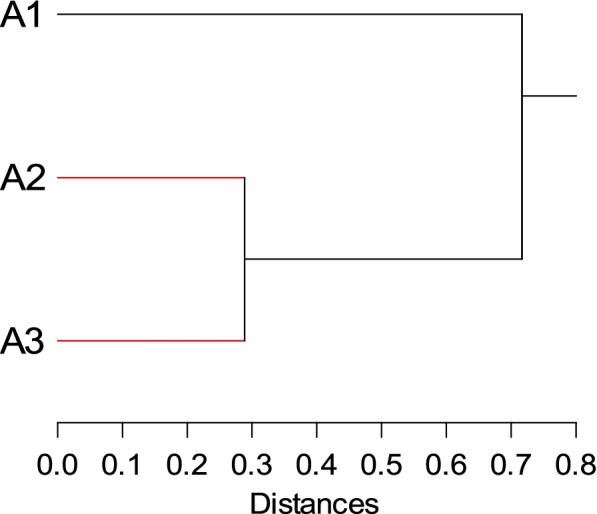
UPGMA dendrogram based on similarity matrix constructed by Euclidean distance metric and Average linkage method for binary data of combined ISSR and SRAP markers for three *A. maurorum* genotypes

Table 6 compared the performance between ISSR and SRAP analyses. Thirty-six amplification products were detected with 16 monomorphic, 20 polymorphic, and 7 unique bands generated by all ISSR and SRAP primers. SRAP primers had the maximum polymorphism percentage of 64%, compared to ISSR markers which had a polymorphism percentage of 36.36%. The total ISSR and SRAP polymorphism percentage was 55.55%. As indicated in Table 7, the maximum similarity index (0.939) was reported between *A. maurorum* at sites A2 and A3, followed by 0.652 at sites A1 and A2. However, the lowest similarity (0.578) was recorded at sites A1 and A3. To elucidate the genetic relationships among *A. maurorum* genotypes, the dendrogram based on SRAP and ISSR data was constructed (Fig. 9) and divided the samples into 2 main clusters. One cluster included *A. maurorum* at A1 site, while the other was sub-clustered into 2 groups separated by 0.3 genetic distance between *A. maurorum* plants at A2 and A3 sites.

**Table 7:** Similarity index for three *A. maurorum* genotypes using ISSR and SRAP analysis

	A1	A2	A3
A1	1		
A2	0.652	1	
A3	0.578	0.939	1

## Discussion

Metal bioaccumulation in plants is a major source of concern as the metal concentrations can surpass WHO-recommended safety levels, posing health and environmental risks to humans and environment [41]. Plants have developed sophisticated signalling systems, such as the ‘universal’ cascade, which can be used to monitor their reactions to HM stress [42]. Signalling channels are in charge of regulating these processes, which consist of a reception step (stimuli perception), a transduction step (intracellular and extracellular signal amplification) and, lastly, a response step (enzymatic or non-enzymatic) [43, 44]. Research studies should fine-tune these mechanisms to make plants safer for consumption through understanding the mechanisms used by plants to respond to HMs.

Fe is the most common element among the numerous metals examined in the groundwater samples of El-Wahat El-Bahariya [45]. In the present study, Fe and its related metals in the soils caused stress to the wild xerophytes growing in this deposit. Different tissues of *A. maurorum* showed different biochemical, ultrastructure and molecular genetic responses to HMs. Distinct variations were recorded in the mining area and other nearby locations in relation to environmental stresses, including aridity, HM toxicity and salinity.

EC and pH are important abiotic factors for determining plant establishment and colonisation in the zones degraded by mining operations, and could influence the metal ion biosorption process efficiency [46]. The soil water extracts were mildly alkaline at the studied sites, and there was a substantial salinity at A3. However, soil samples associated with plants at site A1 had a significantly lower pH than other sites. Higher soil pH levels result in an increased retention and lower HM solubility. A high level of HMs in the soil could suggest a similar concentration in plants, which could pose a significant risk to human health [47].

The presence of one HM in the soil and plant can affect the availability of another. In other words, HMs have both antagonistic and synergistic effects [48]. The interrelationship between soil HMs is very complex. However, in the present study, there was a synergistic relationship between Fe, Zn, Cu and Ni; Cd and Co; and Mo, B and Pb. Moreover, Cr demonstrated substantial antagonistic behaviour against most of the observed HMs.

The above-normal metal accumulation in various *A. maurorum* parts could suggest their resistance to HM contamination in mining areas. As a result, creating a plant population around mining sites may assist in reducing the effects of mining by making soil more sustainable, either by lowering metal concentrations or by immobilising pollutants in the soil [49]. The concentrations of iron and other HMs were lower in the soil than that recorded in plant tissues. For example, cadmium and lead levels in sediments were lower than those in shoots. This result is similar to that recorded previously for the roots of *Cynodon dactylon* developing in the Nakivubo tributaries [50]. HMs are not only bio-accumulated in the roots of certain plants, but also translocated from the roots to the shoots of others [51]. Yanqun et al. [52] found that *A. maurorum* accumulated above-normal levels of 5 mg/kg Pb and 10 mg/kg Cu in contrast to soils. *A. maurorum* accumulated HMs in plant parts more abundantly in shoots than in roots. For phytostabilisation, metal-resistant plants with a low metal concentration are preferred. Those plants accumulate HMs in their roots and are therefore weak translocators [53]. As metals are translocated to easily harvestable plant parts, phytoextraction can reduce HM levels in sediments to appropriate levels over time. In the present study, *A. maurorum* was chosen for phytostabilisation of Fe, B and Mo in its roots, while most HMs (Zn, Mn, Ni, Cr, Pb, Cu, Cd and other micro-elements) were translocated into shoot sections at threshold concentrations.

Differences in concentrations exist between organisms and plant components, suggesting their metal absorption capacities [54]. TF values (shoot/root quotient) less than unity indicate that metals have accumulated and are being processed in the root. For all HMs except Fe, Mo and B, TF values of > 1 suggest preferential partitioning of metals in the shoots at the three sites studied. When the TF value is greater than unity, further translocation occurs in the plant shoot, resulting in phytoextraction as a phytoremediation mechanism. Fast growth and a large root system are also preferred [55]. *Alhagi* species have the deepest root system of any plant in terms of proportion. It is a very hardy and destructive plant. Because of its extensive root system, it is very difficult to eradicate existing populations [56]. This was verified by our phytoextraction findings for most of the studied HMs.

The BCF, which is the ratio of metal concentration in plants to extractable metal concentration in the soil, is used to express accumulation quantitatively [57]. The current findings revealed that the BCF was > 1 for Fe, Pb, Co, Zn, Cr and Cd at all locations. It is concluded that *A. maurorum* could be used as bioaccumulative markers for these metals, and that their concentrations in the soil were well-represented by them. Similarly, *Plantago lanceolata* could be utilized as a bioaccumulative indicator not only for Pb and Zn, but also for Cd, according to Dimitrova and Yurukova [58] who studied this plant in polluted and non-polluted areas.

The SCF values assess the plant ability to accumulate HMs in the shoot biomass [59]. The shoots of *A. maurorum* will accumulate more Mn, Pb, Co, Zn, Cd, Ni, Mo and Cu than the roots. However, our findings as well as those of Moreno-Jimenez et al. [60] support the hypothesis that there are gradients of plant-available metal levels in metalliferous soils, which are mirrored in the metal-tolerant individuals gradient [61, 62]. *Cardaminopsis arenosa* was found to be unsuitable for phytostabilisation because it bioaccumulated high levels of Cd and Zn in its shoots, but had better growth cover than *Dudleya caespitosa* in the soil without the metal stabilising amendments [63, 64]. These findings are in agreement with those of *A. maurorum* shoots.

Plants can immobilise HMs by absorption and accumulation [65]. In *A. maurorum*, the RCF was greater than unity for Mn, Pb, Co, Zn and Cd. This indicates HM tolerance, adaptation to soil and environment characteristics, HM absorption capacity and root spatial fitting to pollutants.

Plants synthesize various secondary metabolites as protective mechanisms against environmental stresses such as salinity, drought, HMs and diseases [66]. The antioxidant protection mechanisms help plants defend themselves from damage when they are exposed to contaminated soils. Higher antioxidant enzyme activities and non-enzymatic constituent levels are essential for plants to withstand stress conditions such as metal toxicity. Originally, these were considered osmotic buffers, but in addition to osmotic modification, they also tend to have an essential role in preserving the natural state of macromolecules, most likely through ROS scavenging [67]. This antioxidant defence mechanism includes enzymatic (SOD, CAT, and APX) and non-enzymatic (GSH, proline, alkaloids, carotenoids and phenols) antioxidants that function as scavengers of free radicals [68]. Some biomolecules involved in cellular metal detoxification might have chelating or antioxidant properties, as previously mentioned.

HM exposure elicited antioxidative responses, but the direction of these responses varied depending on the plant, tissue examined, metal applied and metal stress severity [69]. The present study also examined the non-enzymatic and enzymatic detoxification pathways in *A. maurorum* tolerance to HM stress. *A. maurorum* had higher CAT and POD, and a remarkable and drastic increase in PPO activity at site A1 compared with the other sites. H_2_O_2_ detoxification in plants is supported by CAT. Similarly, Sarker and Oba [70] discovered that an increase in CAT and SOD played a role in ROS detoxification in tolerant *Amaranthus tricolour*. There was a strong link between Cu and antioxidant enzymes (CAT, POD and PPO) in this research. In *Carthamus tinctorius*, Mazhoudi et al. [71] concluded that the stimulation of SOD activity in conjunction with CAT appeared to protect against membrane damage as Cu is especially toxic to membranes.

HMs have been linked to lower chlorophyll levels in several plant organisms. Compared with other sites, *A. maurorum* obtained from site A3 showed a substantial reduction in photosynthetic pigments. Similarly, reductions in the levels of photosynthetic pigments have been detected in different plants exposed to HMs [72]. Carotenoids act as antioxidants, protecting the plant from free radicals and photochemical damage. As a result, the fact that carotenoid levels are reduced may indicate that they play a protective role against oxidative stress [73]. According to Naidoo and Chirkoot [74], a decrease in the Chl (a + b) /Carotenoids ratio in *A. maurorum* at different coverage sites can be interpreted as evidence of a decrease in photosystem II photochemical production.

One of the main protective antioxidant mechanisms of plants exposed to HMs is the activation of biosynthesis of secondary metabolites such as flavonoids and alkaloids [75, 76]. Various studies indicate that polyphenolic compounds have a variety of biological impacts in plants [77]. Alkaloids in plants also have antioxidant properties [78]. In contrast to other sites, *A. maurorum* obtained from site A1 showed lower reductions in proline and increases in protein, flavonoids, alkaloids and tannins (measured by the number and area of shoot tanninferous cells). Previous phytochemical analysis of *A. maurorum* extracts revealed the existence of alkaloids, flavonoids, saponins, glycosides, steroids, tannins and anthraquinones as main components [79, 80].

Metal-detoxification ability of intracellular antioxidant enzymes can be improved by non-enzymatically synthesised compounds such as proline [81]. Proline functions as a stable and metabolic osmolyte, cell wall constituent, antioxidant, free radical scavenger and macromolecule stabiliser [82, 83]. Because of its sensitivity to triple stress categories such as salinity, drought and HMs, *A. maurorum* collected from site A3 had the highest proline content in the present study. Plants produce proline as a non-enzymatic reaction to stress imposed by a variety of abiotic and biotic factors such as drought, salinity, HMs and increased radiation [84].

The results showed that well-water irrigation in iron mining areas increased the protein and flavonoid content of *A. maurorum*, suggesting that they are the first to be exposed to HM contamination and could be the first line of protection. In like manner, Ozyazici [85] speculated that the rise in protein content following sludge treatment may be due to higher available soil nitrogen levels. During metabolism, plants develop complex secondary metabolites. Several of these metabolites could expel free radicals from the organism under stress, however, the metabolites ability to scavenge ROS decreases [86, 87]. *A. maurorum* collected from site A1 had a lower reduction in DPPH free radical scavenging effects than that collected from the other sites. A3 site subjected to various types of stress, had the highest percentage (74.63%). HMs (Cr, Cd and Pb) were applied to MS-medium and *Brassica rapa* seeds were allowed to germinate according to Siddiqu et al. [88]. The in vitro plantlets were collected after germination and tested for DPPH scavenging activity. Control plants that had not been exposed to HMs had a substantially higher activity (87.06%). These HMs, especially Cd, have been found to not only restrict plant growth but also influence antioxidant activity. In contrast, Sulaiman [89] observed that leaf extracts of *A. maurorum* had a high free radical scavenging activity of about 95%, while flower extracts had a free radical scavenging activity of 82%. Moreover, Dhaniya and Parihar [90] showed high antioxidant potential, with 73.30% for the leaf and 88.1% for the stem extracts of *A. maurorum*.

Excess Cu has also been shown in numerous studies to encourage and stimulate the production of fenton-type ROS, resulting in an increase in MDA and dityrosine as oxidative damage biomarkers [91]. In *A. maurorum*, MDA showed a significant linear correlation with increasing metal concentrations, especially Cu. The effects of HMs on the content of MDA and photosynthetic pigments in bean seedlings (*Phaseolus vulgaris* L.) grown in Hoagland solution were investigated by Zengin [92]. MDA levels in HM-treated plants increased dramatically, while chlorophyll content in seedling leaves decreased.

Plants have developed a variety of mechanisms to combat HM toxicity. The main one is the chelation of metals at the intracellular and intercellular levels by forming phytochelatin or metallothionein metal complexes, followed by HM ions removal from sensitive sites and vacuolar sequestration of ligand–metal complexes [81]. GSH serves as a first line of protection against metal toxicity, complexing metals before induced phytochelatin synthesis reaches effective levels [93, 94]. *A. maurorum* showed a lower reduction of reduced GSH in the analysed region in this study. Ishikawa et al. [95] established that HM exposure results in significant depletion of GSH. This is a common response triggered by an increase in GSH consumption to produce phytochelatins. On the other hand, increases in the levels of non-enzymatic antioxidants in AMF-inoculated and calcium-treated plants suggested that they play a role in strengthening the antioxidant protection mechanism that results in continued development [96].

In the present study, most of the studied HMs consumed by the plant were mainly stored in the shoots, and *A. maurorum* had developed anatomical adaptations to cope with metal concentrations in its tissues. Anatomical adaptations were observed in the leaves and stems of *A. maurorum*, despite the presence of higher concentrations of HMs in leaf tissues. We may infer from our findings that *A. maurorum* is a xerophytic species with the thickest stem epidermis and thickened adaxial and abaxial epidermis of leaves. The number, location and distribution of tanninferous cells in the leaves and stem of *A. maurorum* collected from site A3 were increased. Increased tannin content is thought to be one of the non-enzymatic protective mechanisms against HMs in this case. The presence of sunken stomata in the epidermis of both leaves and stems is a water-saving technique. This research supports the findings of Awmack and Lock [27] who reported that anatomical tests of *A. maurorum* stems revealed a thick epidermis and a poorly constructed cortex. Vascular bundles are positioned radially from the centre outward. The xylem is well constructed, allowing significant amounts of water to be transported from the ground without pith. Gomes et al. [26] also stated that *Brachiaria decumbens* exhibited adaptive properties for survival in HM-polluted soil, implying that the species should be investigated further as a possible restorer. The thickened adaxial and abaxial epidermis found in the species may be a technique to reduce water loss by transpiration, which could explain the increased leaf blade turgor shown in the contaminated plants. Leaf curling is a technique for reducing surface transpiration and maintaining stomata in a humid microclimate to avoid dryness [26, 97].

It is important to report that the genes associated with the glutathione metabolism pathway (*6PGD, G6PDH* and *GST*) revealed higher expression levels. GST is a catalytic enzyme that uses glutathione to function in plant stress tolerance processes [98, 99]. Moreover, *6PGD* and *G6PDH* mediate NADP+ reduction to NADPH and play crucial role in the maintenance of glutathione under stresses [100, 101]. These findings were also in agreement with that reported by Wu et al. [99] who revealed higher expression levels of these genes in *Alhagi sparsifolia* plants grown under stress conditions. The results also indicated that these genes might synergistically regulate the glutathione metabolism in *A. maurorum* plants under stress conditions. The current study also revealed that heavy metal accumulation recorded in the different collection sites modulated the expression levels of *nitrate reductase 1*, *sulphate transporter* and *auxin-induced protein* in *A. maurorum* plants, indicating that *A. maurorum* plants respond to heavy metals accumulation via modulating their molecular mechanisms.

The use of molecular markers to investigate plant genetic homogeneity has been recommended since they would target various portions of the genome [40, 102]. One of the most significant advances in the field of molecular genetics is the use of molecular markers for the detection and assessment of DNA polymorphism [103]. In the present investigation, ISSR and SRAP markers have been successfully utilized to assess the genetic variation and fidelity in *A. maurorum* genotypes grown in HM-contaminated soil. Five ISSR primers produced 11 reproducible bands, 7 of which were monomorphic and 4 of which were polymorphic, whereas 25 amplicons in total with 9 monomorphic and 16 polymorphic with 7 unique bands were generated from 6 SRAP primers combinations. SRAP markers detected a high genetic differentiation in *A. maurorum* than ISSR markers. These results were in agreement with that recorded by Amirkhosravi et al. [38] who reported that 8 labeled inter simple sequence repeat (ISSR) primers generated a total of 243 bands used to screen 22 populations including 110 individuals of *Alhagi* species in Iran. Moreover, Abd El- hak et al. [104] studied the genetic diversity of *Alhagi graecorum* populations using 10 SCoT primers. A total of 140 fragments were amplified among the 25 individuals, with 37 monomorphic and 103 polymorphic fragments. Moreover, Jingade et al. [105] reported SRAP markers revealed a considerable polymorphism rate (67.83%) in Indian coffee (*Coffea arabica* L.). Even though, Agarwal et al. [40] reported that the amplification products of ISSR, SCoT and RAPD were found to be monomorphic across all *A. maurorum* samples.

In the current study, the DI and Rp parameters were utilized to estimate the level of polymorphism, genetic diversity, and informativeness of SRAP and ISSR markers. Furthermore, PIC values assist in establishing primers efficiency in genetic diversity analysis [106]. Their effectiveness was demonstrated by high polymorphism percentage and average number of polymorphic fragments per primer. PIC > 0.5, 0.5 > PIC > 0.25, and PIC < 0.25 are all associated with high, medium, or low loci polymorphisms, respectively [107]. The mean of PIC value (0.16) in the present study suggested that ISSR markers might exhibit low loci polymorphism among *A. maurorum* genotypes, but SRAP markers exhibited a moderate mean of PIC value (0.24). Furthermore, ME1xEM7 primer combination, as well as 49 A, HB-10, and HB-11 primers, had the highest PIC and Rp values, making them the most informative primers for discriminating *A. maurorum* genotypes. The use of SRAP primers with moderate PIC values is a good strategy for assessing genetic diversity among *A. maurorum* genotypes. Furthermore, the average PIC value per primer in this investigation was close to that obtained by Soleimani et al. [108] who used SRAP markers which revealed a polymorphism rate of 53%. The average PIC value of ornamental pomegranate (*Punica granatum* L.) was 0.28. Furthermore, Pakhrou et al. [109] discovered that the PIC value (0.27) and marker index (MI = 10.81) produced by IRAP markers were nearly identical to those produced by ISSRs (PIC = 0.27 and MI = 12) of the argan tree (*Argania spinosa* L.) in Morocco. Our findings corresponded with those of Kumar and Agrawal [110] who discovered that SRAP markers were more effective than ISSR markers in evaluating genetic diversity. As compared to ISSR markers (polymorphism percentage of 14.43%, PIC of 0.10) of Indian *Simarouba glauca* DC, SRAP markers generated higher polymorphism percentage (26.54%) and polymorphic information content (0.14). In the present study, the similarity matrices generated by SRAP and ISSR markers were the highest between *A. maurorum* from sites A2 and A3. Our findings corresponded with those of Amirkhosravi et al. [38] who employed ISSR markers to distinguish between *Alhagi* species in Iran, where *Alhagi* has retrieved two species; *A. graecorum* and *A. maurorum.*

## Conclusion

HM-induced anatomical, biochemical and molecular genetic changes are direct indicators of the eco-physiological effects that these HMs have on the plant environment. *A. maurorum* is an ideal candidate for phytoremediation (phytoextraction and phytostabilisation) of Cr, Pb, Cd, Mn, Cu, Ni and Zn in iron mining ore deposits. *A. maurorum* obtained from site A1 (mining) had decreased levels in photosynthetic pigments, proline, DPPH and reduced GSH, and increased levels in antioxidant enzyme activities and encoding genes, MDA, protein, flavonoids, alkaloids and tannins. ISSR and SRAP markers are effective for assessing genetic variation and for differentiation of *A. maurorum* samples. *A. maurorum* demonstrated adaptive characteristics for survival in HM-contaminated soil, implying that *A. maurorum* should be studied further as a possible restorer of mining deposits contaminated by these HMs.

## Materials and methods

### Study area description

El-Wahat El-Bahariya is a vast topographic depression in the center of the Egyptian Western Desert, about 270 km southwest of Cairo and 180 km west of the Nile Valley. This depression is an interesting location in the Western Desert, particularly for rich iron ore deposits. These ores are found in the northern part of the depression and occupy an area of 11.7 km^2^, with a thickness ranging from 2 to 35 m, with an average of 9 m [2].

Three sites in this region were used in the research: A1, El-Gedida, an iron mining site at a longitude of 29°12′ E and a latitude of 28°27′ N; A2, located 5 km from iron mining operations at a longitude of 29°09′ E and a latitude of 28°29′ N; A3, El-Harra located 10 km from iron mining operations at a longitude of 29°04′ E and a latitude of 28°21′ N.

### Collection and preparation of *A. maurorum* and soil samples

An *A. maurorum* sample was collected and pressed using firm cardboard sheets as an herbarium voucher specimen deposited in the botanical herbarium of the Botany Department at the Faculty of Science, Mansoura University, Egypt (deposition number 38). Confirmed sampling permissions needed to collect this plant material were obtained. The species was identified by Prof. Maha M. Elshamy (Professor of Plant Ecology and Flora at Botany Department, Faculty of Science, Mansoura University, Egypt) following Boulos [8]. Soil samples were collected from each of the three study sites, together with plant samples, in a rooting zone depth of 30 cm. Samples were put in plastic bags, placed on ice, transferred directly to the laboratory, and frozen before analysis. Shoot and root samples were preserved in triplicate for nutrient and HM content determination. Also, some shoot and root samples were fixed for ultrastructure analysis, and leaf samples were kept at − 80 °C for later molecular analysis.

### Determination of soil physicochemical properties

Organic matter was assessed in air-dried soil samples [111]. Electrical conductivity (EC) and pH were determined in soil suspensions (1:5 w/v dilution) with digital conductivity (Systronics-304) and pH meters (Labotronics-LT-1), respectively. Mixed-acid digestion was used to extract HMs from 0.5 g samples of dry soil. Total nitrogen (N) was assessed using the Kjeldahl method. Potassium (K) was determined using a flame photometer (CORNING M410), and phosphorous (P) was spectrophotometrically measured using the molybdenum blue method. Fe, Mn, Pb, Zn, Cd, Cu, Co, Ni, Mo, B, and Cr were digested with sulphuric acid (H_2_SO_4_) and estimated using inductively coupled plasma–optical emission spectroscopy (flame-AAS, GF-AAS, and ICP-AES) [112].

### Bioavailability of heavy metals and nutrient concentrations in *A. maurorum*

HMs were extracted using a mixed-acid digestion process from 0.5 g samples of roots or shoots. Total nitrogen (N), potassium (K), and total phosphorus (P) were analyzed using the same techniques as for soil samples.

Phytoremediation capacity, accumulation, and upward translocation of HMs were assessed by calculating bioaccumulation factors (BCF), root coefficient factors (RCF), shoot coefficient factors (SCF), and translocation factors (TF) as [113, 114] from Eqs. (1), (2), (3) and (4):

1 $$\mathrm{BCF}=\mathrm{HM}\;\mathrm{concentration}\;\mathrm{of}\;\mathrm{plant}\;\mathrm{root}\;\mathrm{or}\;\mathrm{shoot}\;\left(\mathrm{mg}\cdot\mathrm g^{-1}\right)/\mathrm{HM}\;\mathrm{in}\;\mathrm{soil}\;\left(\mathrm{mg}\cdot\mathrm g^{-1}\right)\;\mathrm{for}\;\mathrm{each}\;\mathrm{site}$$

2 $$\mathrm{RCF}\:=\:\mathrm{HM}\;\mathrm{conc}.\;\mathrm{in}\;\mathrm{root}/\mathrm{HM}\;\mathrm{conc}.\;\mathrm{in}\;\mathrm{soil}$$

3 $$\mathrm{SCF}\:=\:\mathrm{HM}\;\mathrm{conc}.\;\mathrm{in}\;\mathrm{shoot}/\mathrm{HM}\;\mathrm{conc}.\;\mathrm{in}\;\mathrm{soil}$$

4 $$\mathrm{TF}\:=\:\mathrm{HM}\;\mathrm{conc}.\;\mathrm{in}\;\mathrm{shoot}/\mathrm{HM}\;\mathrm{conc}.\;\mathrm{in}\;\mathrm{root}$$

### Biochemical analyses

#### Enzymatic antioxidant activities

CAT activity was measured spectrophotometrically as the decrease in absorbance of H_2_O_2_ at 240 nm [115]. POD was measured as previously described Zheng and Van Huystee [116]. Absorbance at 420 nm due to the formation of purpurogallin was used to measure polyphenol oxidase (PPO) activity [117].

#### Non-enzymatic antioxidant levels

Photosynthetic pigments were assessed by treating 2 g of fresh tissue with 50% acetone (v/v) and refrigerating at 4 °C overnight in full darkness. The extract absorbance was then measured spectrophotometrically at 453, 644, and 663 nm against an aqueous acetone blank [118]. Concentrations of pigments were determined, and values expressed as mg·g^**−** 1^ fresh wt. Total protein content of sample extracts was measured spectrophotometrically [119]. Proline content was estimated as reported by Bates et al. [120], and absorbance at 520 nm was read against a toluene blank. Proline content was estimated as 1 μmol proline per gram of fresh weight.

Total flavonoid content was estimated following the colorimetric aluminum chloride method [121]. The absorbance was read at 415 nm. A calibration curve was used to quantify total flavonoid content as quercetin at concentrations of 12.5–100 mg. mL^− 1^ in methanol. The antioxidant capacity of ethanolic extracts, with ascorbic acid as a standard, was calculated using scavenging of the stable 2,2-diphenyl-1-picrylhydrazyl (DPPH) free radical [122]. The solution absorbance was read at 515 nm per minute for 30 minutes. DPPH scavenging activity was determined using Eq. (5) as follows:

5 $$\mathrm{Scavenging}\;\mathrm{activity}\;\left(\%\right)\:=\:\left[\left(\mathrm A\:-\:\mathrm B\right)/\mathrm A\right]\:\times\:100$$

Where A represents the absorbance of control (only DPPH in solution), and B represents the absorbance of DPPH with sample.

Alkaloid content was assessed following Harbone [123]. An orbital shaker was used to extract 2.0 g of dry powders using 50 mL of 10% acetic acid in ethanol for 4 hours at 200 rpm (Panasonic, MIR-S100, Japan). The filtered mixture extract was condensed to one-quarter of its original amount in a water bath. Concentrated ammonium hydroxide was mixed with the extract before the completion of precipitation. The whole suspension was left to settle; the precipitates were taken, washed with dilute ammonium hydroxide, and purified. A trace was then dried, and alkaloid content measured.

MDA was extracted from *A. maurorum* shoots [124] with slight modification. Briefly, MDA was collected from fresh shoot samples (0.5 g) using 5 mL of 0.1% (w/v) trichloroacetic acid (TCA). Extracts were centrifuged for 25 minutes at 3000×*g*. Next, 2 ml of 0.5% thiobarbituric acid (TBA) and 5% TCA were mixed with 3 mL of extract supernatant. TBA reacted with MDA in the acidic medium during incubation in a water bath (95 °C) for 30 min. Reactions were stopped at room temperature, and a microplate reader was used to measure the absorbance of resulting pink solutions (TBA-MDA adducts or reaction products) at 534 nm. MDA content was determined following the formula introduced by Wang et al. [125].

GSH was estimated using high-performance liquid chromatography (HPLC). *A. maurorum* aerial tissues (leaves) were boiled in 3 L of distilled water for 1 hour before drying under reduced pressure. Powder was stored in a clean bottle before use. Reduced GSH was measured in extracts using HPLC. Each extract was purified with a 0.22 μm syringe filter, and 10 μL of the filtrate was injected into a Waters 2690 Alliance HPLC system (Waters Inc., Milford, CT, USA) with a Waters 996 photodiode array detector for sample analysis. A 1 mg/mL GSH aqueous stock solution was prepared, followed by serial dilution to obtain concentrations of 50, 100, 150, 200, and 250 μg/mL. These dilutions were also purified using a 0.22 μm syringe filter and 10 μL of filtrates were injected into the HPLC. Reduced GSH was separated on a C18 Xterra column, 4.6 × 250 mm, with a gradient sustained at a flow rate of 1 mL/min over a run time of more than 10 minutes. Buffer (0.2 M hexane sulphonic acid and 0.01 M KH_2_PO_4_ adjusted to pH 3 with orthophosphoric acid) and methanol comprised the binary mobile phase. Isocratic elution was used for gradient elution from the column (80%:20%). An ultraviolet detector was used to measure reduced GSH at 210 nm. Chromatographic peaks were established by comparing analytical retention times to the retention of reference compounds.

### Semi-thin ultrastructure of *A. maurorum* shoots

One to two mm^3^ leaf or stem tissue samples were incubated overnight at 4 °C in 0.1 M phosphate-buffered saline (PBS, pH 7.4) with 2.5% (v/v) glutaraldehyde and 2% paraformaldehyde [126]. After washing with PBS for 15 minutes, samples were dehydrated using graded ethanol dilutions and embedded in Epon pure resin overnight at 4 °C. Resin was polymerized at 70 °C for 3 days, and 0.5 and 0.1 μm section was cut with a knife using Leica EM UC7 ultramicrotome (Leica Mikrosysteme GmbH, Austria). Semi-thin 0.5 μm section was mounted on glass slides, stained with 1% (w/v) Toluidine Blue O with 1% (w/v) sodium borate for 5 minutes, viewed using an Olympus CX31RTSF model microscope (Olympus Corporation, Japan), photographed with ToupCam (X Cam Full HD camera), and examined using Image J (version 1.50i) software. Two representative images from a total of 10 images per species were digitized.

Stem parameters quantified stem cuticle thickness, chlorenchyma thickness, epidermis thickness, vascular bundle area, phloem area, xylem area, and pith cell diameter. The midrib was used as a reference point to measure leaf anatomical parameters. Epidermal thickness was measured on abaxial and adaxial margins, as was mesophyll. Gland size was also measured. Most measurements used the straight tool of Image J (https://imagej.nih.gov/ij/) on multiple consecutive sections (*n* = 25 aggregates/leaf). Vascular bundle area and gland size were measured using the freehand selection tool.

### Molecular genetic analyses

#### Expression analysis of stress-related genes

Quantitative real-time PCR analysis was conducted to assess the expression levels of six genes (*GST*, *G6PDH*, *6PGD*, *nitrate reductase 1*, *sulfate transporter*, and *auxin-induced protein*) in *A. maurorum* plants collected from the study sites, A1, A2, and A3. Briefly, total RNA and cDNA were prepared from plant tissues with RNeasy Plant Mini and Reverse Transcription kits (Qiagen), respectively. Using QuantiTect SYBR Green PCR kit (Qiagen), PCR reactions were prepared and performed in a total volume of 25𝜇l under previously described specific amplification conditions [99]: 50 °C for 2 min and 95 °C for 10 min; 40 cycles of 95 °C for 15 sec and 60 °C for 45 sec; 1 cycle of 95 °C for 15 sec, 60 °C for 15 sec, and 95 °C for 15 sec. Gene-specific primers were utilized for PCR amplification (Table 8) [99]. *Actin* was chosen as a reference gene. Relative expression levels were calculated using the method of Livak and Schmittgen [127].

**Table 8:** Primers used in the quantitative RT-PCR analysis [99]

Gene name	Primer sequence (5′-3′)
*GST*	F: TCTTGGAGAACGCTCTTGGT
	R: GATGTCATGCTTGAACGCCTC
*6PGD*	F: GGGTTGTGGGGTTGGCTATT
	R: CCCTCTGAGCCTGAACAAGG
*G6PDH*	F: GGAGTCTCAAGGTGAAGCCT
	R: GGTGAAGTGCTTAGGGAGACA
*Nitrate reductase 1*	F: GCTCAAGCGCTGTGGAATTT
	R: GCCTGAGACAGCAAGGTACA
*Sulfate transporter*	F: TGCTTGGGTATATTCAGGCTGG
	R: GTGCTTCACCACTGCTACAAC
*Auxin induced protein*	F: TGGCTCTACCCCTCAGAGAT
	R: CATCGACAGAACACGGAAGC
*Actin*	F: GCGGGAAATTGTTCGTGACA
	R: AAGAACTTCTGGGCAACGGA

#### Genetic diversity analysis using ISSR and SRAP markers

Five ISSR primers and six SRAP primer combinations were tested to assess polymorphism and genetic diversity. Five distinct ISSR primers (49A, HB-9, HB-10, HB-11, and HB-13) of 10–17 nucleotides were chosen. Forward and reverse SRAP primer combinations were also used (ME1xEM7, ME1xEM8, ME2xEM6, ME2xEM7, ME5xEM6, and ME5xEM8) (Table 6). DNA was extracted from young *A. maurorum* leaves using a DNeasy plant Mini Kit (http://www.biobasic.com) and stored at − 20 °C for PCR amplification.

ISSR amplification reactions used a total volume of 30 μl, consisting of 2 μl of each primer, 25 mM MgCl_2_, 1 U Taq polymerase, 2.5 mM of each deoxynucleotide, and 25 ng of genomic DNA. ISSR primer amplification was programmed in an automated thermal cycle (model Techno 512) for one cycle at 94 °C for 1 min, followed by 45 cycles of 1 min at 94 °C, a specific annealing temperature of 57 °C for 1 min, and a final extension for 2 min at 72 °C. Finally, the reaction was kept at 72 °C for 10 min. Amplification products were stained with ethidium bromide, separated on 1.5% agarose gels, and photographed under UV light.

Amplification of SRAP primers was completed using a DNA Thermal Cycler (model Techno 512, UK) with cycling parameters of 2 min of denaturation at 94 °C, 5 cycles of 3 steps: 1 min of denaturation at 94 °C, 30 sec of annealing at 35 °C, and 30 sec of elongation at 72 °C. The annealing temperature was increased to 50 °C for a subsequent 35 cycles, and extension used one cycle of 5 min at 72 °C. PCR products were separated on 2% agarose gels in 1X TBE buffer (89 mM Tris2, mM EDTA, 89 mM Boric acid) at 115 V for 2.5–3 hrs. A 100–1500 bp standard DNA ladder was used for quantification. For binary data, only SPAP and ISSR amplified bands that were clear, strong, distinct, and reproducible were analyzed. Other parameters, such as the total number of polymorphic bands, diversity index, and polymorphic information content, were estimated using the equations of Gorji et al. [128]. The ability to distinguish among *A. maurorum* genotypes was assessed by determining the resolving power (Rp) using the formula of Prevost and Wilkinson [129].

### Statistical analysis

Metal content and physiological, biochemical, anatomical, and molecular genetic data were collected in a randomized complete block design with three replications. Data distributions were tested for normality and equality of variance. ANOVA was used to determine the statistical significance of differences among all factors with post hoc tests set by SPSS (version 22.0, IBM Corp., Armonk, NY, USA); treatments were considered as independent variables. Data are expressed as means and standard errors. A *p*-value of ≤0.05 was considered statistically significant, and data are depicted in figures using SigmaPlot 14.0 (Systat Software, Inc., CA, USA). Pearson correlation coefficients were determined for metals in the soil at study sites and biochemical parameters from associated specimens of *A. maurorum* (Origin Lab Inc., Hampton, USA). Further statistical analyses, including a heatmap of Pearson correlation coefficients among metal concentrations, biochemical parameters, and the ultrastructure of *A. maurorum* shoot samples, were used to identify metals affected by biological activity in shoot samples [130]. Systat Software (Inc., San Jose, CA, USA) was used to analyze genotype correlations in the dendrogram.

## Data Availability

All data generated or analyzed during this study are included in this published article.
